# Urban objects detection from C-band synthetic aperture radar (SAR) satellite images through simulating filter properties

**DOI:** 10.1038/s41598-021-85121-9

**Published:** 2021-03-18

**Authors:** Deepak Kumar

**Affiliations:** Amity Institute of Geoinformatics & Remote Sensing (AIGIRS), Amity University, Sector 125, Gautam Buddha Nagar, Noida, Uttar Pradesh 201303 India

**Keywords:** Environmental impact, Environmental sciences

## Abstract

Satellite-based remote sensing has a key role in the monitoring earth features, but due to flaws like cloud penetration capability and selective duration for remote sensing in traditional remote sensing methods, now the attention has shifted towards the use of alternative methods such as microwave or radar sensing technology. Microwave remote sensing utilizes synthetic aperture radar (SAR) technology for remote sensing and it can operate in all weather conditions. Previous researchers have reported about effects of SAR pre-processing for urban objects detection and mapping. Preparing high accuracy urban maps are critical to disaster planning and response efforts, thus result from this study can help to users on the required pre-processing steps and its effects. Owing to the induced errors (such as calibration, geometric, speckle noise) in the radar images, these images are affected by several distortions, therefore these distortions need to be processed before any applications, as it causes issues in image interpretation and these can destroy valuable information about shapes, size, pattern and tone of various desired objects. The present work aims to utilize the sentinel-1 SAR datasets for urban studies (i.e. urban object detection through simulation of filter properties). The work uses C-band SAR datasets acquired from Sentinel-1A/B sensor, and the Google Earth datasets to validate the recognized objects. It was observed that the Refined-Lee filter performed well to provide detailed information about the various urban objects. It was established that the attempted approach cannot be generalised as one suitable method for sensing or identifying accurate urban objects from the C-band SAR images. Hence some more datasets in different polarisation combinations are required to be attempted.

## Introduction

Satellite remote sensing is being used over several years for constant mapping and monitoring of various earth features including urban objects^1,2^. These methods are always desired over manual survey methods due to additional efficiency like time, accuracy, spatial coverage^1,3,4^. Several space exploration agencies including Indian Space Research Organization (ISRO)^3^ in India and other international spaces agencies including the European space agency (ESA), the National Aeronautics and Space Administration (NASA)^4^ have pushed various optical satellites like IRS series, Landsat^3,6,7^, MODIS Terra, Aqua, Sentinel 2 along with synthetic aperture radar (SAR) satellites such as RISAT-1, Sentinel 16. During recent years, optical remote sensing observations are getting obsolete due to their incapability to penetrate through dense cloud cover^6,7^. Nowadays these are broadly used for several planning and monitoring activities. But in many of the situations, there is a requirement for remote sensing technology to assess the state of the ground reality during the cloud cover and extreme weather events^8^. Observation of the surface conditions from radar technology has a different perspective and it offers several scenarios for feature detection^1,9,10^. But obtaining any radar images have multi-faceted issues including data quality, cost of data acquisition, data preprocessing or correction methods, and augmented distortions. These datasets get effected with several induced errors and these require data processing^1,11^. The major part of the processing methods emphasizes the reduction of augmented distortion or noise from the acquired SAR images.

In this concern, the European Commission has also tried to establish the Copernicus Programme, which created a new paradigm shift towards the availability and accessibility of data to deliver various earth observation services with the help of satellites and in situ data under six thematic Copernicus services^5,12,13^. This programme has become the world’s largest space free and open access data provider of the satellite data acquired by various Sentinel satellites. The improved spatial resolution and high revisit frequency over the globe enable them useful for a wide range of applications^9^. Sentinel-1 satellite constellations acquire Synthetic Aperture Radar (SAR) data in single or dual polarization with a revisit time of 6 days. The Sentinel-1 provides continuous day and night (i.e. allows the acquisition of imagery regardless of weather and illumination conditions) synthetic aperture radar (SAR) imagery over the globe with the help of two polar-orbiting satellites (Sentinel-1A and Sentinel-1B) and these satellites operates in C-band at a centre frequency of 5.405 GHz^9,14^. The major application of Sentinel-1 imageries includes monitoring of sea ice, oil spills, marine winds, waves & currents, land-use change, land deformation and emergency responses in cases of floods and earthquakes^15^. One of the six thematic Copernicus services provides data through the Copernicus Open Access Hub with two product formats like Ground Range Detected (GRD) and Single Look Complex (SLC)^16,17^. Sentinel-1 level-1 GRD products consist of focused SAR data that has been detected, multi-looked and projected to ground range using an Earth ellipsoid model^18^.

Usually, any SAR images have inherent salt and pepper influence, which are referred to as speckles and these are responsible for damaging the quality of the image. Consequently, it results in tougher interpretation of spatial features^19^. These occur due to random constructive and destructive interference within each resolution cell of the image^18,20^. So, before any application these radar images, it needs to be pre-processed and analysed after images enhancements and noise or distortion suppression to reduce or remove undesirable noise^6,9^. The preservation of edge information the SAR images requires reduced or minimum speckle-noise^6,19^. Spatial or Speckle noise reduction approach is performed either by spatial filtering or Multi look processing operations to the SAR datasets. This processing approach uses kernels of different shapes & sizes in form of a window having a magnitude dimension of 7$\times$7, 9$\times$9, 11$\times$11, 13$\times$13, and 17$\times$1710. These kernel traverses over the spatial extent of images on a pixel to pixel basis to enhance the spatial features with enriched details^21,22^. In some of the cases, the radiometric and textural aspects show the low efficiency in feature class discrimination due to the existence of speckle noise in the images^23,24^. But due to diverse characteristics of filters, every filter has their effect on the various objects and this property is being used by these filters for the preservation of spatial objects in the images and information on edges of the features (which is one of the vital segment of feature identification or recognition), sharpening ramp edges to enhance the precise details for reduction of speckle-noise by averaging neighbourhood values^19^.

The study on urban objects identification requires the preservation of quantitative and qualitative details of the images25. Therefore, this research mainly focuses on the presentation of several image enhancement methods with special emphasis on spatial filtering for suppression of noise from the acquired SAR images from several platforms^25–27^. It is always interesting to pursue research on the suppression or lowering of speckle noise. The current work aims for (a) detailed methodology for pre-processing of dual-polarized C-band synthetic aperture radar (SAR) images, (b) assessment of speckle-noise filters, (c) Simulation and evaluation of speckle filters properties for image enhancement.

Several research reports that the spatial filtering or Multi look operation of SAR images with different combination provides better interpretation capability, as the effect of speckle-noise on an image contributes to non-interpretation^20,28,29^. The key focus of the work is aligned for reducing the speckle noise effects based on statistical values or parameters^6,9^. It will affect the interpretation, classification and application of radar images for various purposes. There is a continuous requirement for identification of an urban feature due to the continuous transitions in the urban characteristics^26,30^. Synthetic-aperture radar (SAR) has a wide capability for such objects detection for further applications. The work extends the characteristics of SAR images for spatial object detection in urban scenarios to deliver an improved object detection method based on backscatter values of various features from C-band dual-polarized SAR datasets for further comparison of dissimilar characteristics for better accuracy or precision for object detection.

## Materials and methods

### Study area

**Figure 1:**
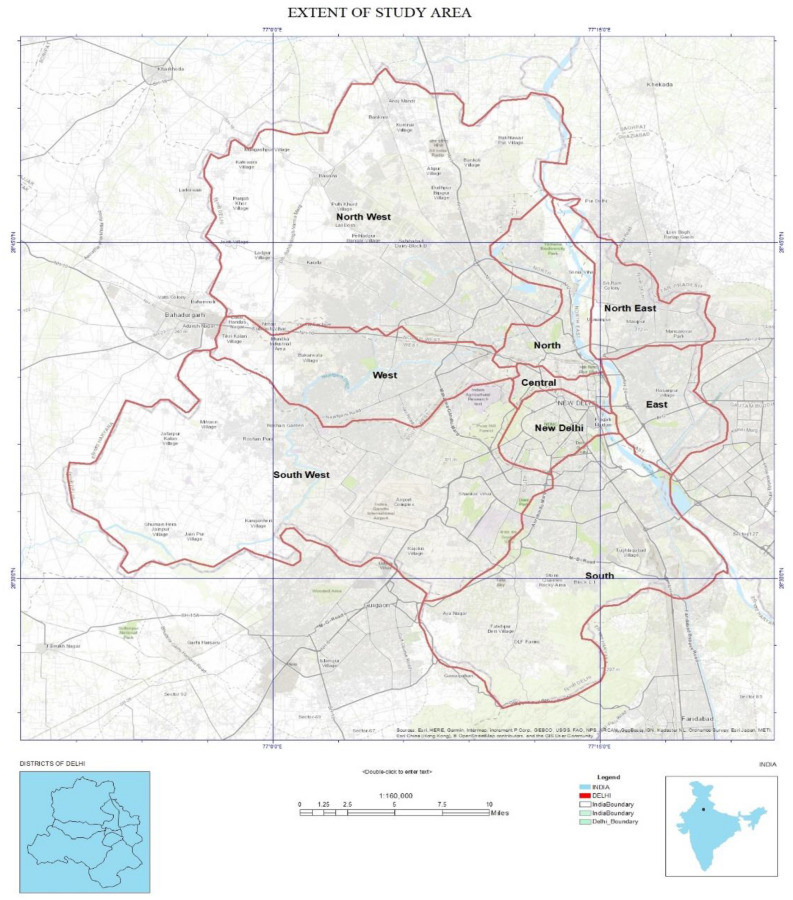
The extent of the study area (Map created with Arc Desktop 10.x Ver.).

The sample study is performed over the capital city of India (i.e. Delhi) for detailed analysis. The city is situated in the northern part of India. This city ranks 2nd populated city in India and is among the top 10 megacities in the world with an increasing prominence^31,32^. The city covers an aerial extent of about 1482 square kilometres and has an average elevation of 216 m above the mean sea level. The city encompasses the cultural blend. The exact location of the study area (i.e. Delhi)^33^ can be seen through Fig. 1. Being a major political, educational, and economic nucleus, the city of Delhi is also experiencing unprecedented pressure on the urbanization^34^. The selected city area was chosen to facilitate the diverse variety of urban features. The complete city can be sub-divided into three major zones like Municipal Corporation of Delhi (MCD), New Delhi Municipal Council (NDMC), and Delhi Cantonment Board (DCB)^35,36^. The River Yamuna is a prominent source of water in this area and the city experiences an average annual rainfall of about 61 cm (i.e. occurs during the months of July to September is monsoon season). The city area experiences the features of the semi-arid climate with sandy soil, which contributes relatively low soil moisture^37^.

### Datasets/databases

The present work utilizes the free datasets available from Sentinel-1 satellite system (comprises of two concurrent satellites Sentinel 1A and Sentinel 1B). Each of the satellite carries C-band SAR sensors to provide data at 5 m spatial resolution with a repeat cycle of 12-day (for each satellite) at an altitude of 693 km with a swath of 400 km.

Sentinel-1A/B C-band SAR is capable to transmit a signal in either horizontal (H) or vertical (V) polarisation and it can receive in both H and V polarisations. This causes the generation of dual polarisation products from this satellite system. The concurrent synchronization of these two satellite orbits causes a 6-day repeat cycle to deliver a single or dual-polarization C-band SAR datasets in Single Look Complex (SLC) or Ground Range Detected (GRD) formats^38^. There is no phase information available with GRD product formats and are available in three modes such as full resolution (FR), high resolution (HR), and medium resolution (MR). GRD products contain the sensed amplitude and it can be multi-looked to reduce the impact of speckle in the image10.

**Table 1:** Summary of metadata for Sentinel-1A/B C-band synthetic aperture radar (SAR) images.

Parameters/properties	Details
Mission	Sentinel-1A
Acquisition mode	IW
Antenna pointing	Right Facing
Average scene height	257.08719127738925
Orbit Cycle	152
Incidence near	30.76721279098475 Degree
Incidence far	46.21031450776563 Degree
Pass	DESCENDING
Product type	GRD
Range looks	5.0
Transmit (T${}_{x)\ \ }$& Receive (R${}_{x}$) Polarisation	VH, VV
Sampling rate	64.34523812571427 MHz
Range spacing	10.0 m
Azimuth spacing	10.0 m
Pulse repetition frequency (PRF)	1717.128973878037 Hz
Radar frequency	5405.000454334349 MHz
Rage bandwidth	56.5 MHz
Azimuth bandwidth	327.0 Hz

### Methods and methodology

C-band SAR datasets acquired from Sentinel-1 satellite orbits are more informative in terms of spatial resolution with high revisit frequency, which enables them for a wider range of applications. For converting C-band dual-band SAR image digital pixel values to the equivalent radiometrically calibrated backscatter values, it is required to process the required calibration information available with the metafile. A calibration vector offers a scheme to convert image intensity values to equivalent gamma nought values. Some work requires GRD or SLC datasets with a standard set of preprocessing for various applications. A generic pre-processing workflow is created within the Sentinel application platform (SNAP) framework for pre-processing of GRD data sets. The pre-processing workflow consists of processing steps designed for reduction of error propagation in each subsequent processes, and are described in following subsections.

The Sentinel-1 GRD level-1 SAR images are required to be processed to equivalent backscatter coefficient ($\upsigma ^{\circ }$) images in decibels (dB) scale. This backscatter coefficient represents the target backscattering area (radar cross-section) per unit ground area. It is required to be converted into dB as 10*log${}_{10}\upsigma \mathrm {{}^\circ }$, as it can vary by several orders of magnitude. It measures whether the surface backscatters from the incident microwave radiation are preferentially away from the SAR sensor dB $\mathrm {<}$ 0) or towards the SAR sensor dB $\mathrm {>}$ 0).

This scattering behaviour depends on the physical characteristics of the surface, objects or terrain, and primarily it is effected by the geometry of the surface features and their electromagnetic characteristics. These mentioned phenomena can be recognised with the following pre-processing steps *(as implemented by the* SNAP Toolbox) to derive the backscatter coefficient for each pixel of the SAR image.

### Preprocessing of Sentinel-1 GRD datasets in SNAP toolbox

**Figure 2:**
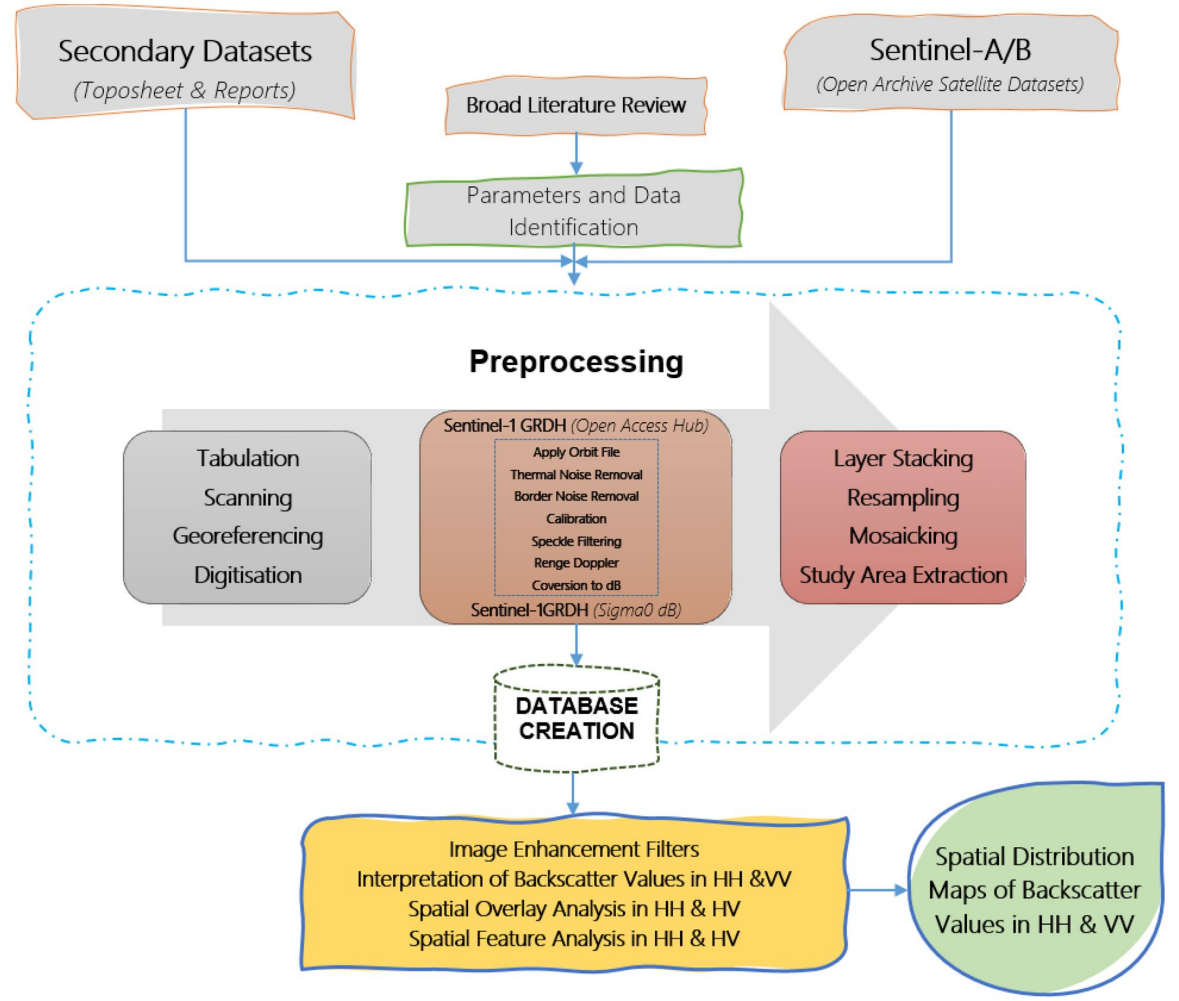
Flowchart for preprocessing of Sentinel-1 GRD datasets in SNAP Toolbox.

Complete chain of preprocessing sequence for Sentinel-1 GRD datasets in SNAP Toolbox is shown in Fig. 2 and it includes the following key steps:

1. **Apply orbit file**: This step updates orbit metadata with a restituted orbit file. The information on-orbit state vectors contained within the metadata information of SAR products are generally not accurate. The precise information for each satellite orbits are resolved after several days of the satellite pass and these are available days-to-weeks after the product generation. The application of precise orbit allows the automatic download and update of the orbit state vectors for each SAR scene to provide an accurate satellite position and velocity information.
2. **Thermal noise removal**: The intensity of Sentinel-1 images are disturbed by additive thermal noise in the cross-polarization channel. Effects of the thermal noise are required to be removed from inter-sub-swath texture to normalize the backscatter signal within the entire Sentinel-1 scene. SNAP toolbox provides the functionality for the thermal noise removal from Sentinel-1 datasets. So, this operation removes additive noise from sub-swaths in multi-swath acquisition modes.
3. **GRD border noise removal work as** an operator in SNAP toolbox and it was designed to remove low-intensity noise and invalid data at the scene edges of the datasets. It is required to adjust the sampling start time, to compensate for the effect of earth’s curvature. As a generation of level-1 products is effected by azimuth and range compression, which leads to radiometric artefacts at the image borders.
4. **Radiometric calibration:** This process transforms digital SAR data pixel values to equivalent radiometrically calibrated SAR backscatter intensity images. It computes backscatter intensity with the help of sensor calibration parameters provided with the metadata information of GRD datasets. It converts image intensity values into equivalent sigma naught values. To generate radiometrically calibrated SAR backscatter to the nominal horizontal plane. The values of Sigma specifies the strength of echo in terms of the geometric cross-section of a distributed target. The values of the sigma naught vary with respects to variation in the incidence angle, wavelength, and polarization including surface properties of the scattering surface.
5. **Speckle filtering: **SAR images contain granular noise, namely known as speckles, and these are caused due to the interference of waves returned from scatterers. This speckle or spatial filtering method is applied to enhance the quality of the image with a reduction of speckles in the image.
6. **Range Doppler Terrain correction/Terrain correction(Orthorectification):** The terrain corrections are applied to compensate the distortions caused due to earth’s geometry. There are multiple geometric distortions like as foreshortening and shadows caused by topography and these are required to be corrected for each location of the SAR image pixel. It translates the data from ground range geometry to $\upsigma$ $^\circ$ with the help of SRTM 30 m DEM or the ASTER DEM for high latitudes (greater than 60$^\circ$ or less than −60$^\circ$) and it does not consider the terrain conditions into account.
7. This is typically performed through the range-Doppler terrain correction operation with the help of a digital elevation model. It characterizes the processed image as the best possible real-world image.
8. Conversion to dB converts unit less backscatter coefficient to equivalent dB (decibel) on a logarithmic scale.
9. Simulation of Filters Properties on C-band SAR Image in SNAP Toolbox

**Figure 3:**
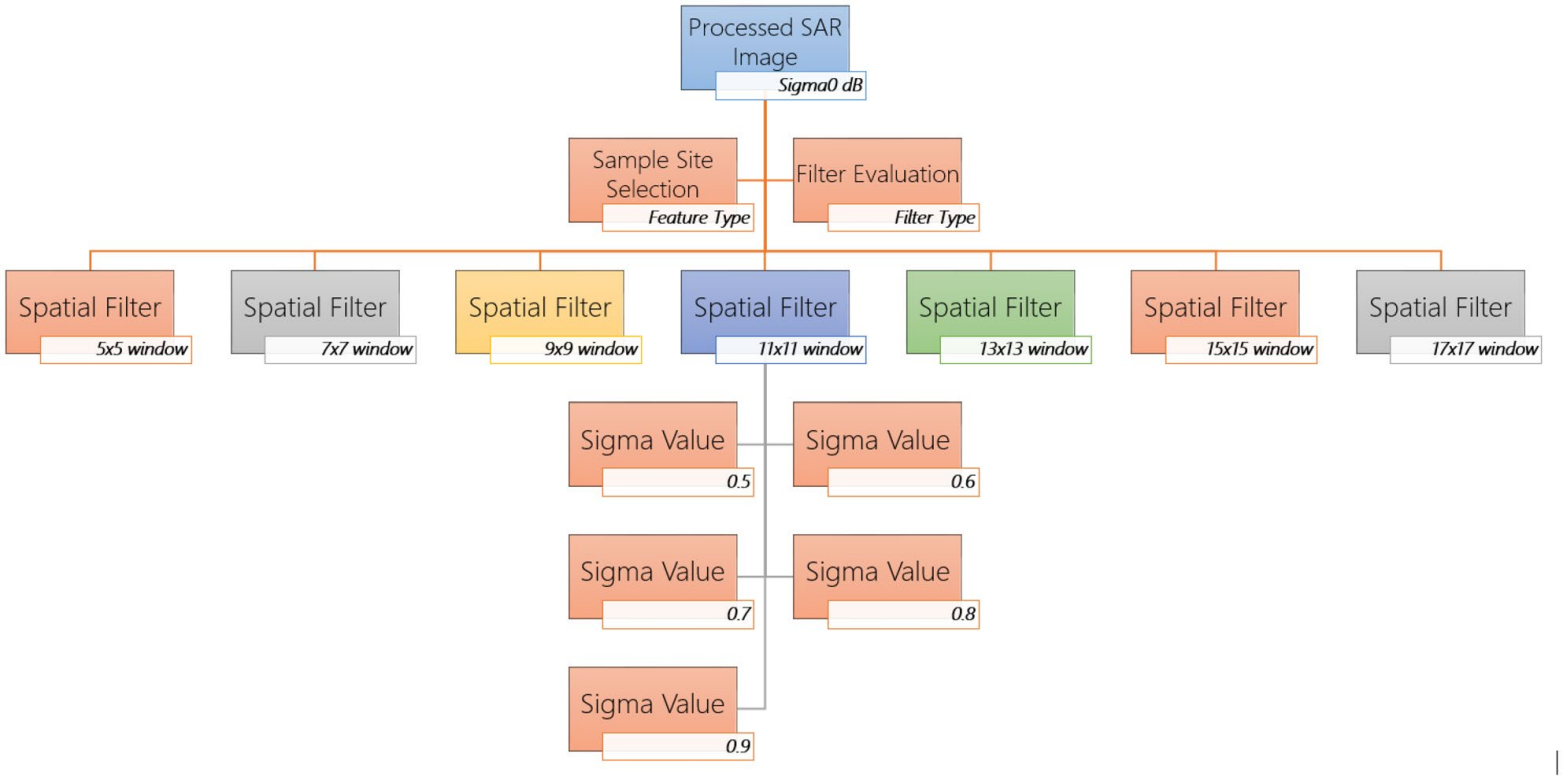
A conceptual framework for simulation and evaluation of filters properties.

The preprocessing workflow for identification of the suitable speckle or spatial filter can be done through simulation of multiple speckle filter properties with its parameters like window size and sigma values. SNAP toolbox facilitates a variety of filters in form of single product speckles filter operator such as ‘Boxcar’, ‘Median’, ‘Frost’, ‘Gamma Map’, ‘Lee’, ‘Refined Lee’, ‘Lee Sigma’, and ‘IDAN’. These operators can be well evaluated for their peculiar properties in object detection or identification (especially about urban features). Figure 3 displays the proposed conceptual framework for simulation and evaluation of filters properties with SNAP Toolbox for understanding the role of speckle or spatial filters for object detection/identification from a SAR image.

It summaries the broad stages of data preprocessing. As SAR datasets are usually sensed with a varying viewing angle greater than 0 degrees, which results in the addition of some distortion associated with the side-looking geometry. But the application of speckle or the spatial filter at an early stage of SAR data processing reduces the chance of speckle propagation in ongoing processes (i.e., terrain correction or conversion to dB). In many of the instances, speckle filtering is not suitable especially in the identification of small spatial feature, feature structure or image texture, as it could confiscate such information. Hence, it becomes very much crucial in application and selection of speckle filters at different levels. Normally, we consider refined Lee filter as superior in compared to other single product speckle filters for better visual interpretation due to its capability to preserve edges, linear features, point target and texture information of the image. But recently, multi-temporal speckle filters have been developed to reduce speckle, which takes advantages of multiple SAR observations.

## Results and analysis

Sentinel-1 datasets are captured in C-band with a dual-polarization mode at range resolution of 5 m and azimuth resolution of 20 m. The backscatter information accessed in VV, VH polarization mode for the analysis as an independent variable. The acquired datasets are better in terms of spatial resolution with high revisit frequency for a wider range of applications. The generated outputs at every step are being shown in the following section with discussion.

### Outputs from preprocessing of C-band SAR images

#### Effect of orbit file update operation on the SAR image

**Figure 4:**
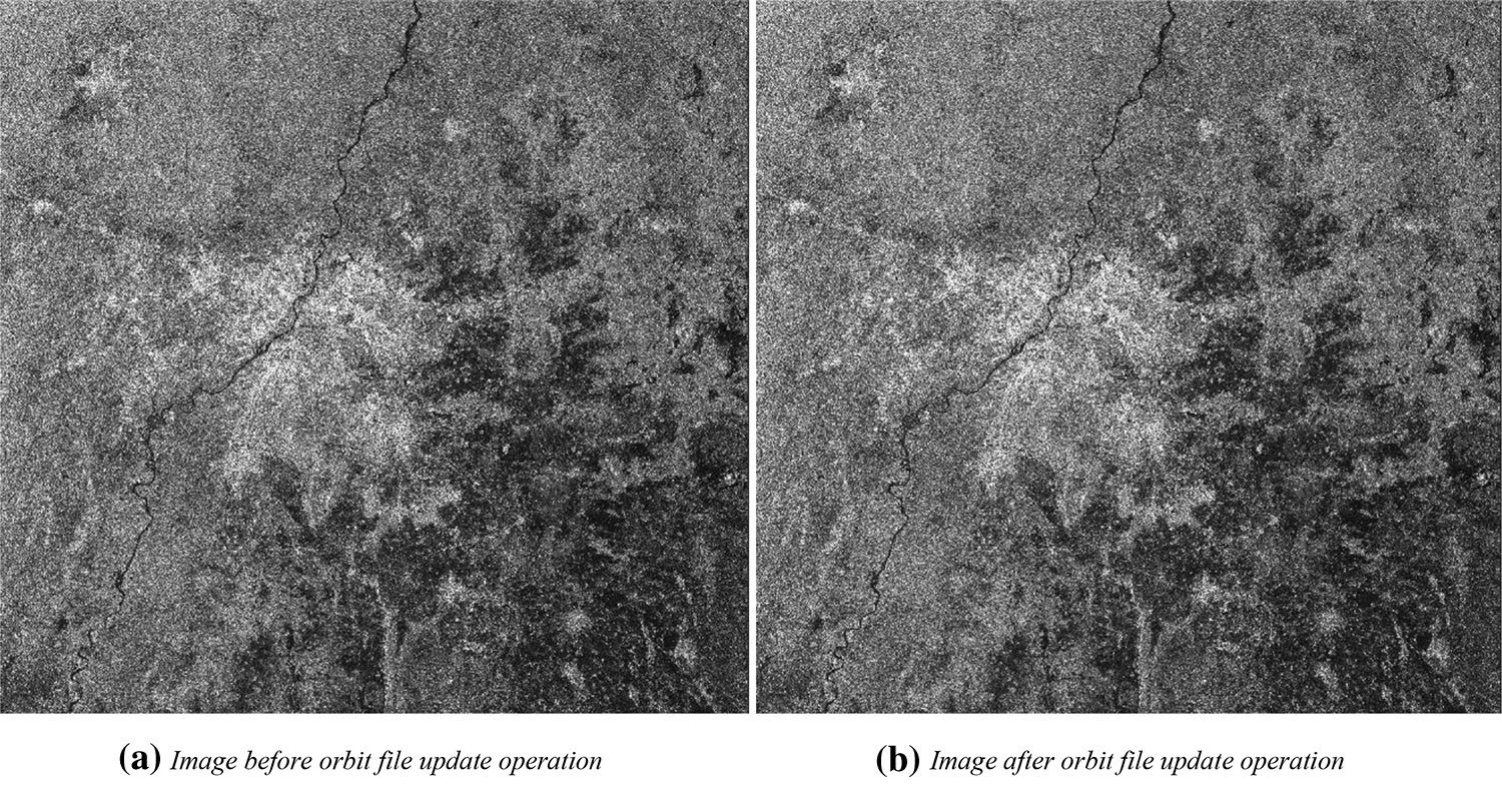
Effect of orbit file update operation in VH polarisation.

The image generated after the application of the orbit file can be seen in Fig. 4a,b. Figure 4a displays the image before the orbit file update operation, and Fig. 4b exhibits image after orbit file update operation for each polarization. The application of a precise orbit update of the orbit file to each of the SAR scenes provides an accurate position and velocity information of the satellite.

#### Effect of thermal noise removal on SAR image

**Figure 5:**
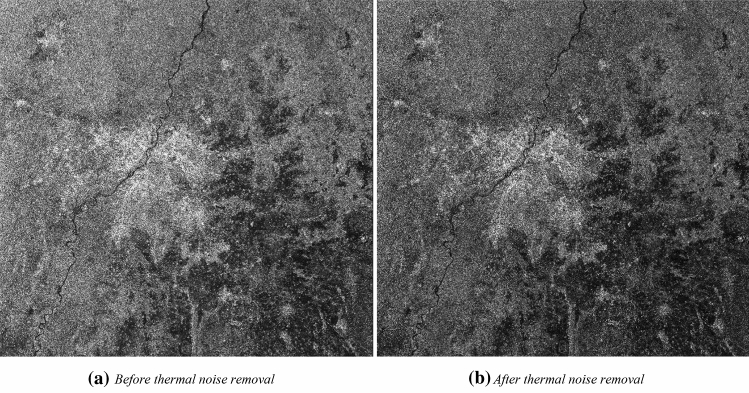
Effect of thermal noise removal in VH polarisation.

The thermal noise removal operator available in SNAP toolbox is applied to the orbit corrected Sentinel-1 datasets. Figure 5a,b expresses the images before and after thermal image removal operation to the images.

It can be observed that thermal noise operation removal decreases the influence of noise in the inter sub-swath texture due to the normalization of the backscatter signal within the entire scene.

#### Effect of border noise removal on SAR image

**Figure 6:**
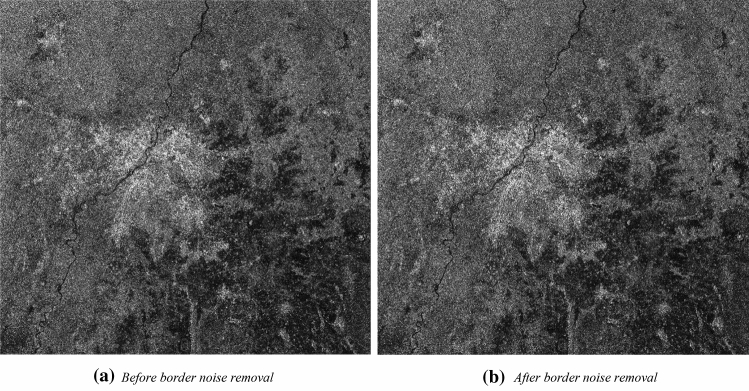
Effect of border noise removal in VH polarisation.

This operation characteristically eliminates the low-intensity noise and invalid data from the scene edges. It also compensates the influence of Earth’s curvature giving radiometric artefacts at the image borders. Figure 6a shows the image before the use of border noise removal operation and Fig. 6b shows the image generated after border noise removal operation. It can be observed that Fig. 6b has reduced border noise.

#### Effect of radiometric calibration on SAR image

**Figure 7:**
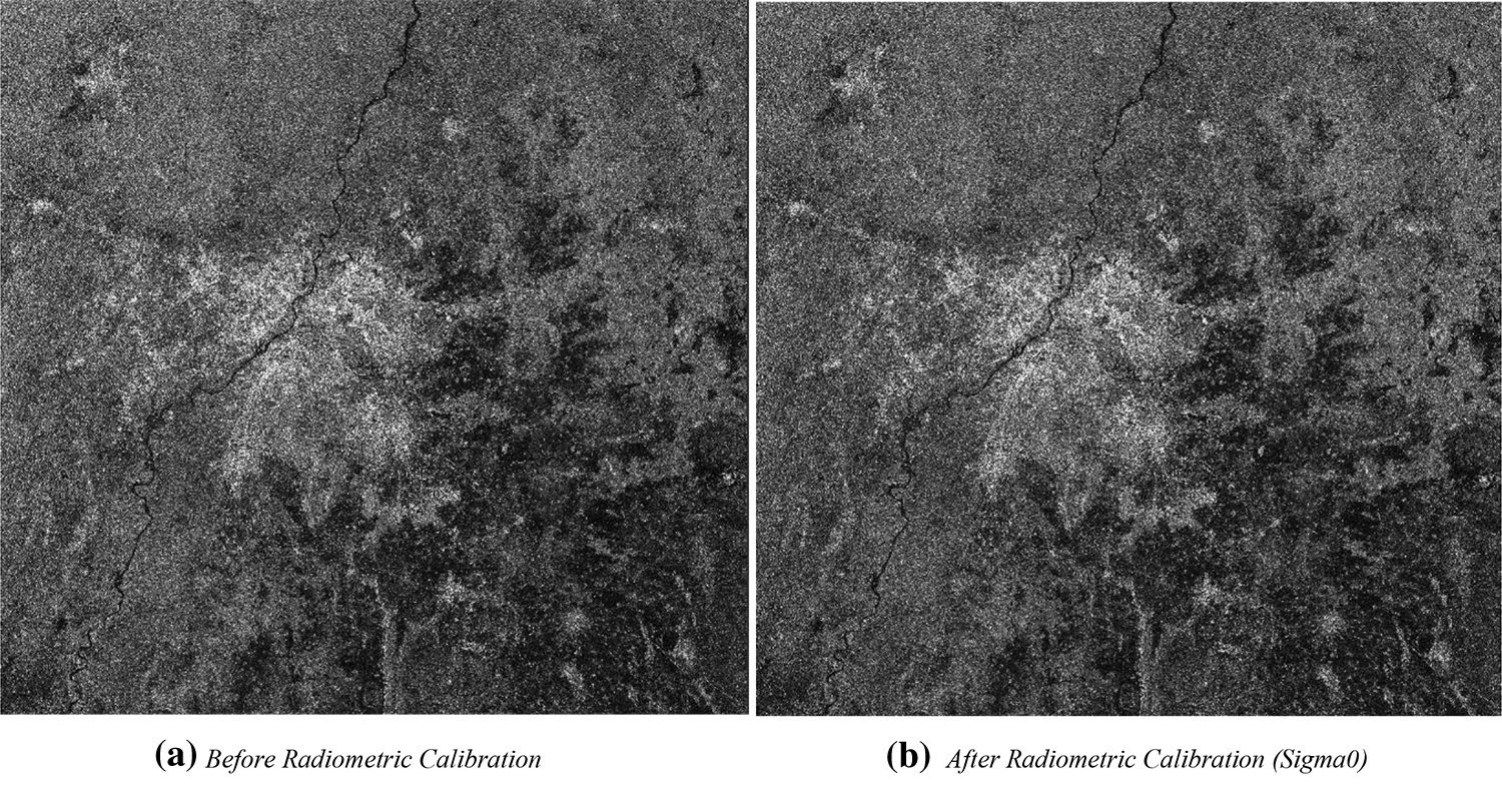
Effect of radiometric calibration in VH polarisation.

The effect of conversion on the intensity of the image into equivalent sigma naught image can be seen in Fig. 7a,b. Figure 7b shows the image generated after the radiometric calibration operation, whereas Fig. 7a shows the prior image. It can be seen in Fig. 7b that, it visualizes the sigma naught values to generate radiometrically calibrated SAR backscatter. These variations in sigma naught values specify the strength of reflection or backscatter from the target or surface features. The variation of sigma naught values occurs due to variation of incidence angle, wavelength, polarisation, and surface scattering properties.

#### Effect of multi-look (ML) operation on SAR image

**Figure 8:**
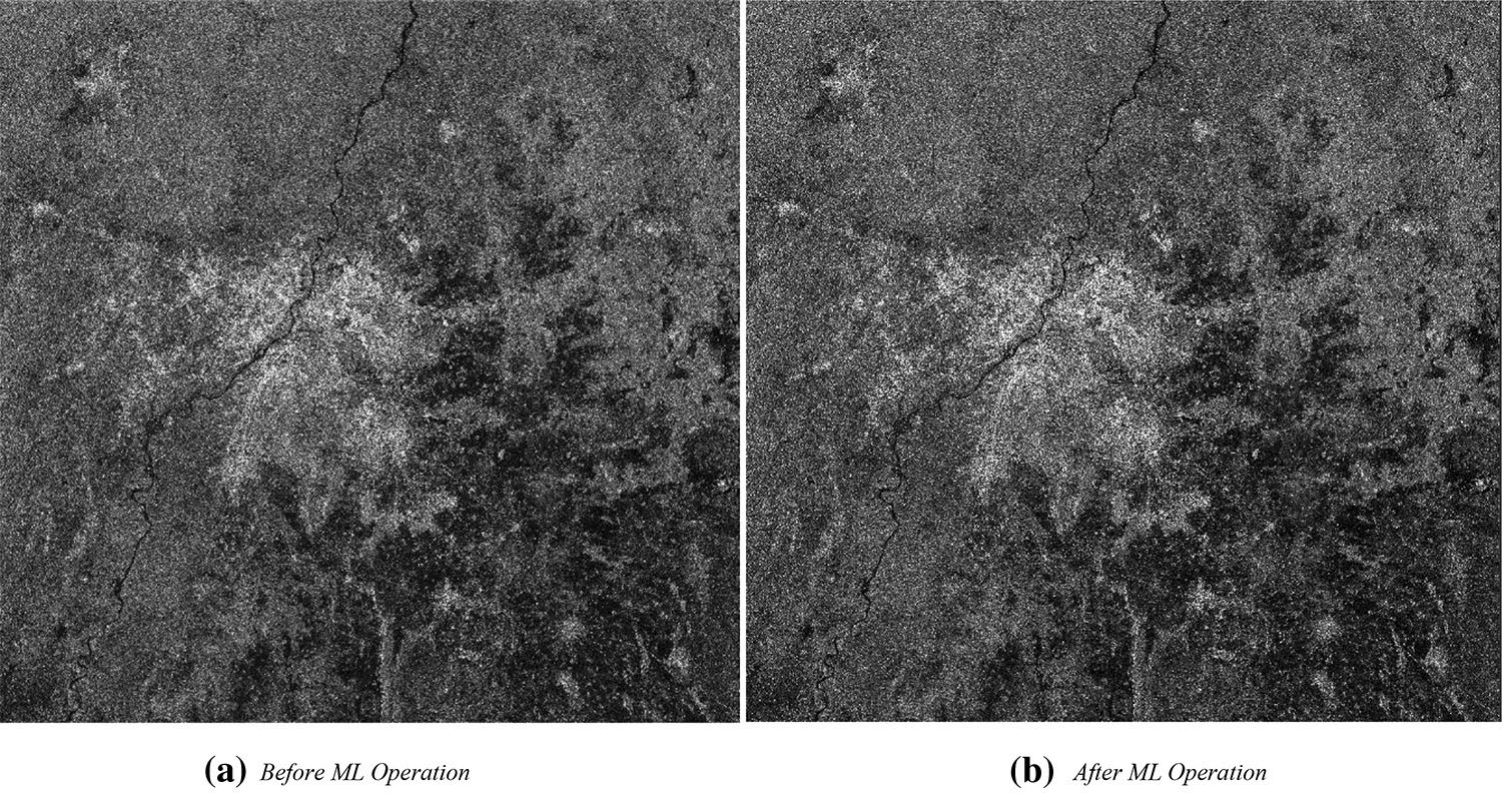
Effect of Speckle filter in VH polarisation.

SAR images are affected due to the presence of speckles in the image and these occur due to redirected echoes from the targets. Figure 8a displays the speckle effect on the image, which makes it difficult for interpretation due to the presence of noise in the image. The influence of speckle-noise can be minimized with the use of multi-look operation to the image. Figure 8b is generated after the application of the usual speckle reduction operation.

#### Effect of range-Doppler terrain correction on SAR image

**Figure 9:**
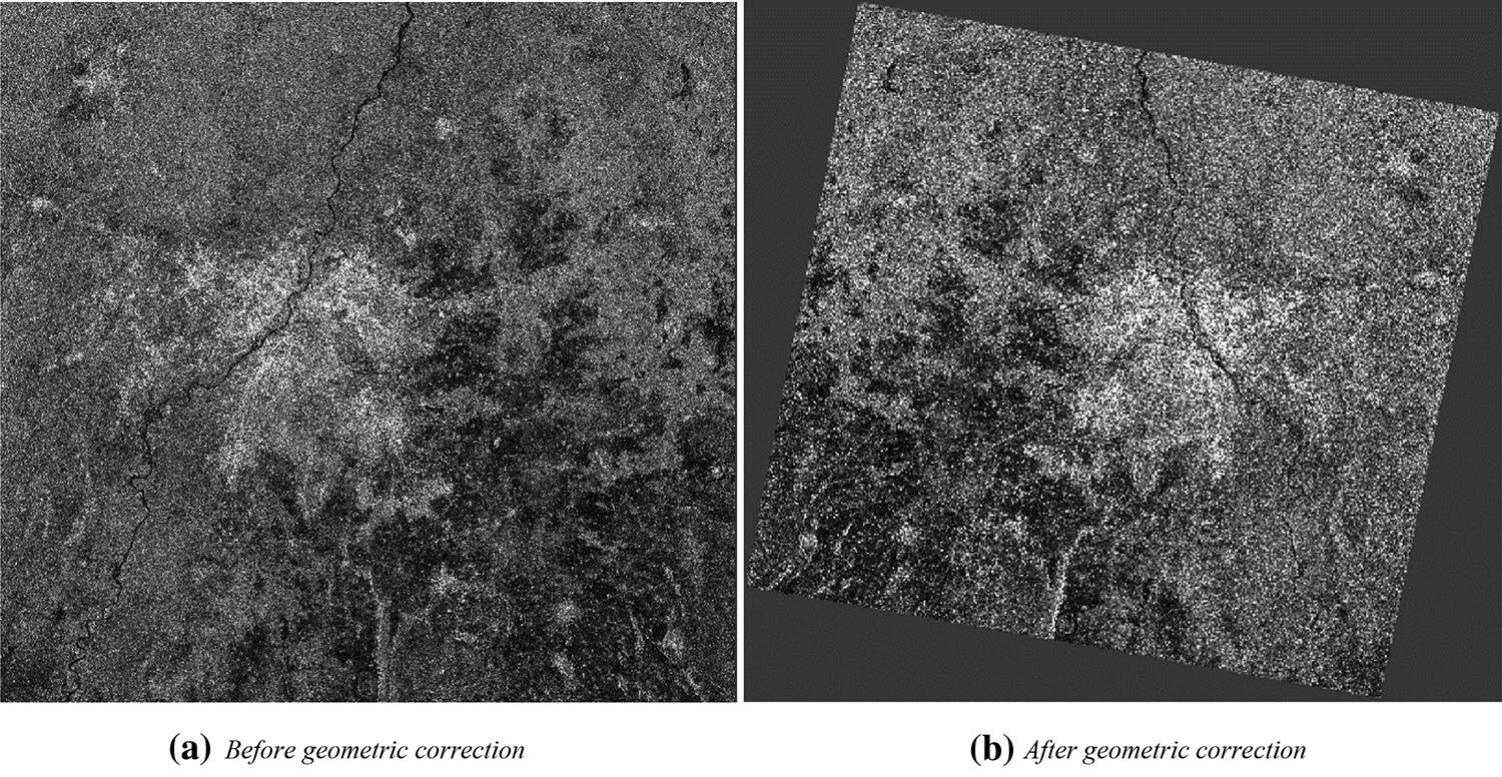
Effect of geometric correction/range Doppler terrain correction in VH&VV polarisation.

There is an impact of geometric distortions on the SAR images due to characteristics of the sensor, platform and object. Range Doppler terrain correction process reduces the geometrical distortions present in the SAR image. Figure 9a shows the image under influence of the geometrical distortions, and Fig. 9b displays the image after the range-Doppler terrain correction operation. These geometrically corrected remotely sensed SAR images are used for the generation of numerous thematic layers and maps.

#### Auxiliary outputs generated during the geometric correction step

Due to topographical variations in the radar scene induced due to the tilt of the satellite sensor can distort or affect the quality of the SAR images. Image is not directly acquired at the sensor’s Nadir location, so it will add some distortion in the scene. Terrain corrections operations compensate these distortions for the very accurate or precise geometric representation of the processed image close to the real world. In the course of the geometric correction operation on the image, the following additional outputs get generated to envisage the surface condition of terrain seen by the radar sensor.

**Figure 10:**
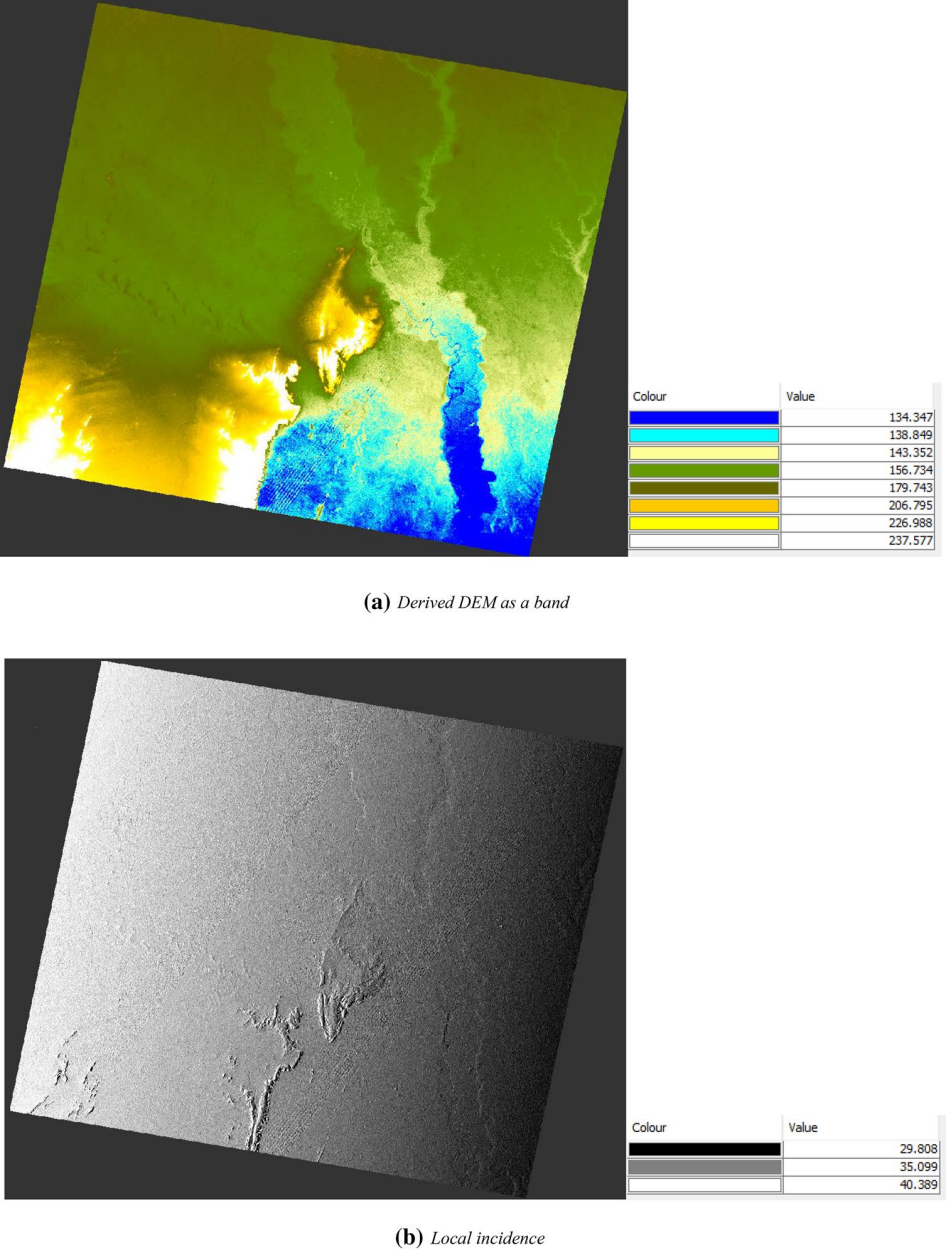
Auxiliary outputs created during geometric correction step.

The auxiliary derived information from the radar sensor during the geometric corrections show that the general elevation in the derived DEM (digital elevation model) ranges from 115.207 to 415.411 m. But the colour ramp classification indicates that the minimum elevation is 134.347 m (shown as blue colour in Fig. 10a) and the maximum elevation is 237.75 m (shown as a white colour in Fig. 10b).

Figure 10a exhibits the local incidence angle image being generated to show the angle between the normal vectors of the backscattering element. The local incidence angle image (as shown in Fig. 10a shows the angle between the normal vector of the backscattering element (i.e. vector perpendicular to the ground surface) and the incoming radiation vector (i.e. vector formed by the satellite position and the backscattering element position). Figure 10a articulates that the values of the local incidence angle are 29.808 degrees, 35.099 degrees, and 40.389 degrees. The minimum and maximum value of the local incidence angle lies between the ranges of 16.866 degrees to 65.234 degrees. The projected local incidence angle from DEM has the angle between the incoming radiation vector (as defined above) and the projected surface normal vector into a range plane. Here range plane is the plane formed by the satellite position, backscattering element position and the earth centre.

#### Image conversion to dB

The preprocessing workflow includes a step for the conversion of the image into an equivalent dB scale for better visualization and interpretations is an integral part of the preprocessing. The unit less backscatter coefficient is converted to equivalent dB with the help of a logarithmic scale conversion.

**Figure 11:**
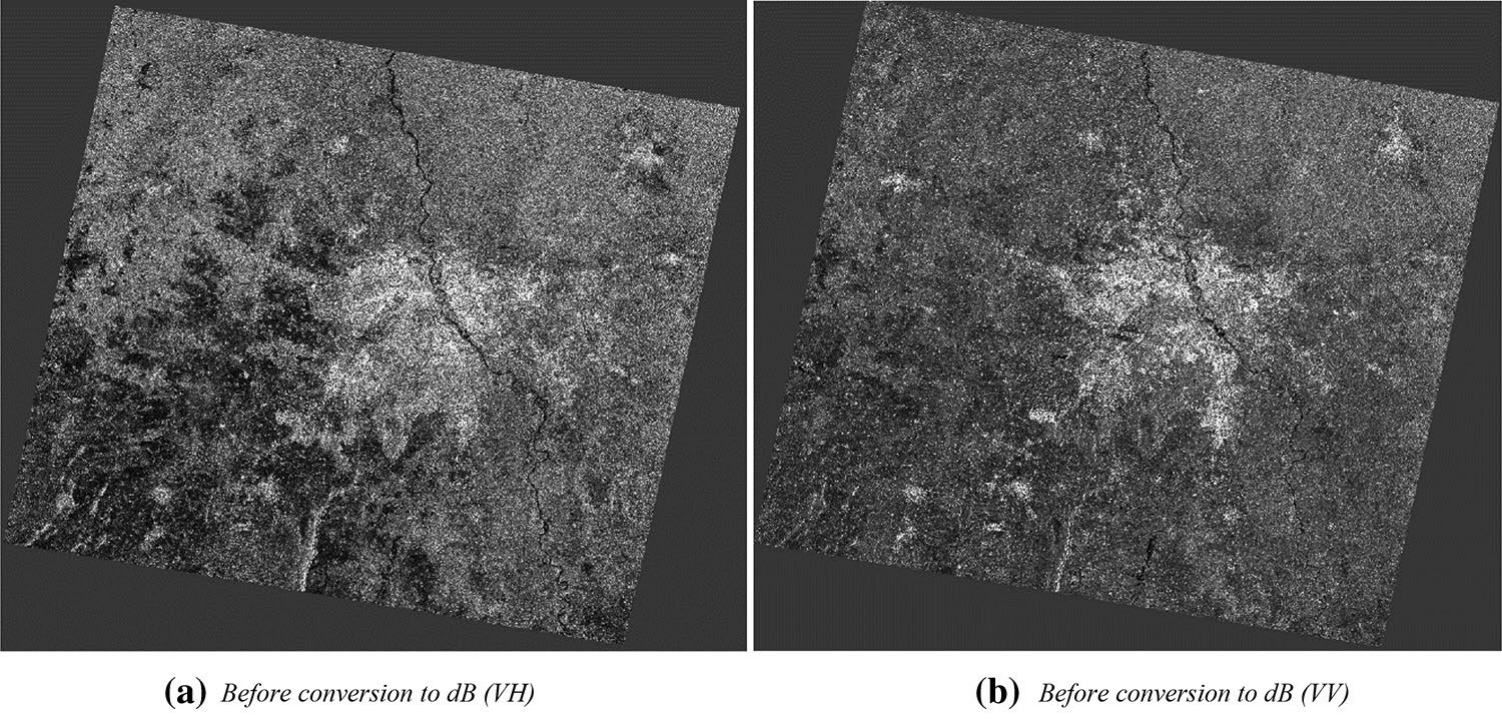
Conversion of the image to dB.

**Figure 12:**
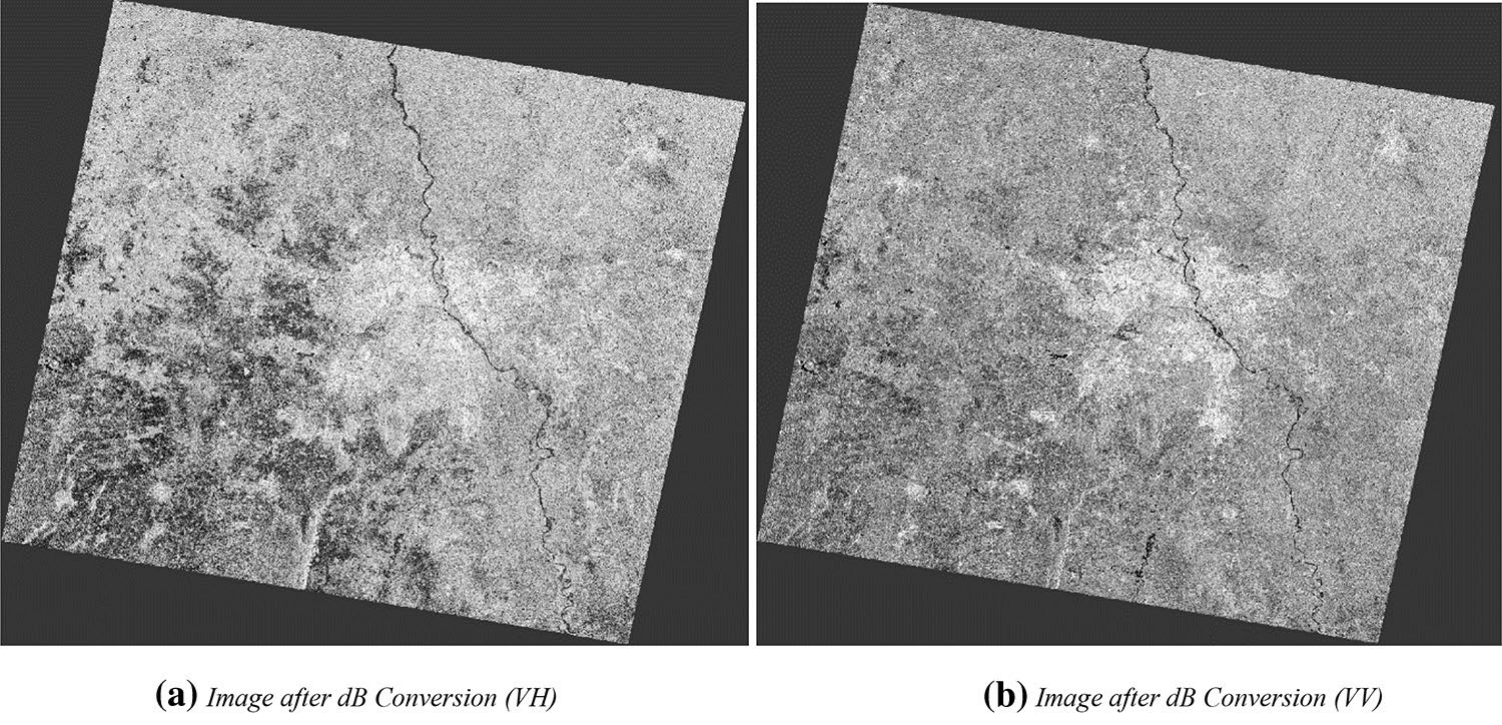
Converted output dB image in dual polarisation.

Figure 11a,b exhibits the image generated before the image in linear scale, whereas Fig. 12a,b displays the terrain corrected in Sigma0 in dB (decibel) scale in VH and VV polarisations. It can be seen that all man-made features are well highlighted due to strong backscatter returns from these surfaces. It can be observed that converted equivalent dB image indicates more highlighted features or objects for interpretation in the images compared to earlier image and more visually more prominent. Figure 12 shows the split view of Fig. 11 in corresponding dual-polarization mode with dissimilar interpretation capability. In the VH polarised image, water features show the dark colour tone, consequently VH polarised image envisages the river water bodies more predominantly in compared to VV image. It enables easier detection of water bodies from the C-band radar image due to no back-scattering behaviour of the water surfaces.

### Original colour composite image of pre-processed C-band SAR image

**Figure 13:**
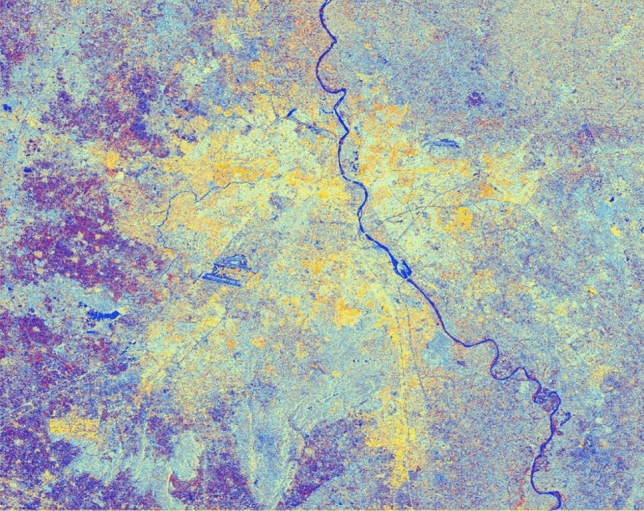
Colour composite image (Red: VV, Green: VH, Blue: VH/VV) of the Sentinel-1 dual-polarization C-band data with application of the speckle/spatial filters.

The corresponding SAR colour composite (CC) image generated after stimulation of filter properties for better interpretation for the features can be seen in Fig. 13. It can be seen from Table 13 that there is variation in backscatter values in all the bands as per their surface properties.

Figure 13 exhibits the dual-pol ratio colour composite without the application of any filter on the pre-processed SAR image.

### Outputs generated after stimulation of filters properties on C-band SAR image

#### Simulated output with a window size of 5 and sigma value of 0.5 for C-band SAR image

**Figure 14:**
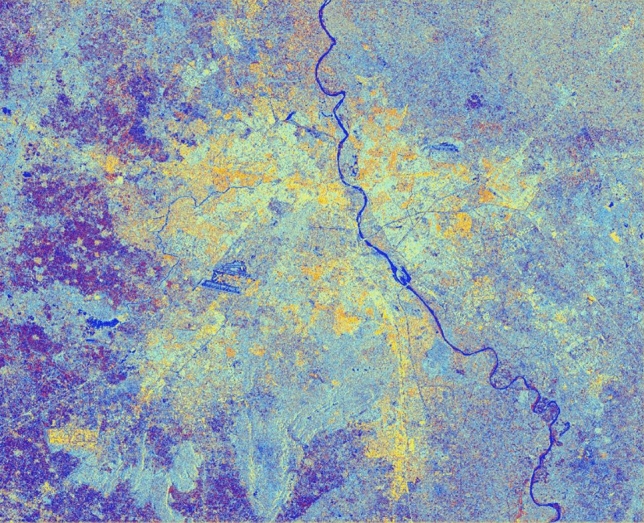
Colour composite image (Red: VV, Green: VH, Blue: VH/VV) of the Sentinel-1 dual-polarization C-band data in with a window size of 5 and sigma value of 0.5.

It can be observed that all urban objects/features are prominently highlighted as per their surfaces roughness or smoothness properties. The yellow shade exhibits the presence of urban settlement in a clustered manner. Similarly, Fig. 14 presents the generated image after simulation of filter properties for better interpretation and understanding of the study area with a blend of diverse window sizes and constant sigma value of 0.5.

#### Simulated C-band SAR Image with a window size of 7 and sigma value of 0.5

**Figure 15:**
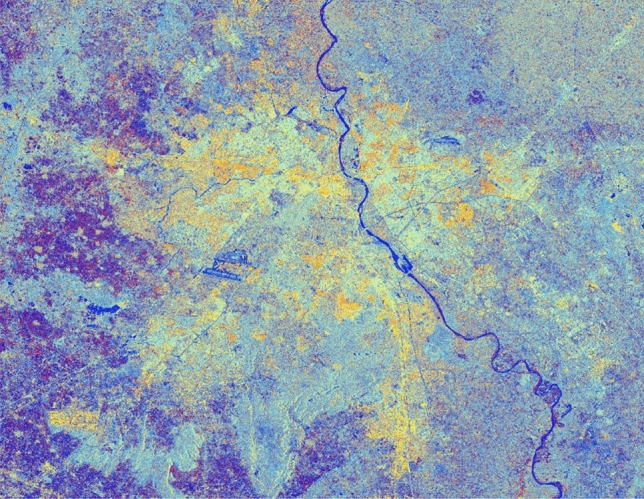
Colour composite image (Red: VV, Green: VH, Blue: VH/VV) of the Sentinel-1 dual-polarization C-band data in with a window size of 7 and sigma value of 0.5.

**Figure 16:**
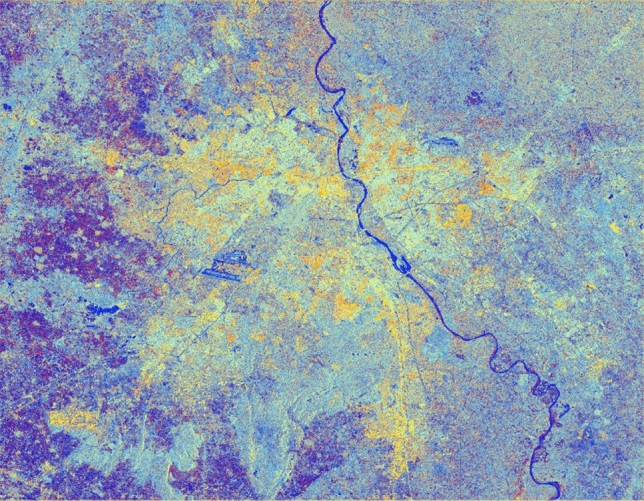
Colour composite image (Red: VV, Green: VH, Blue: VH/VV) of the Sentinel-1 dual-polarization C-band data in with a window size of 9 and sigma value of 0.5.

**Figure 17:**
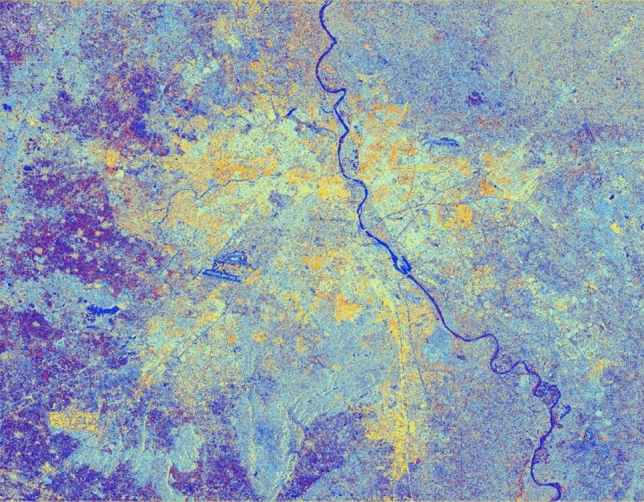
Colour composite image (Red: VV, Green: VH, Blue: VH/VV) of the Sentinel-1 dual-polarization C-band data in with a window size of 11 and sigma value of 0.5.

**Figure 18:**
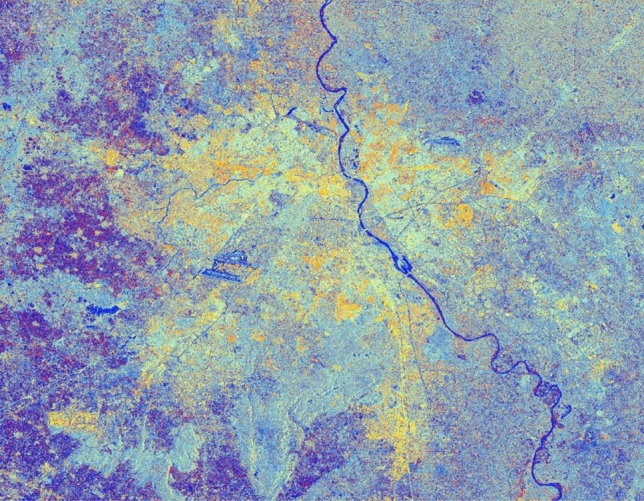
Colour composite image (Red: VV, Green: VH, Blue: VH/VV) of the Sentinel-1 dual-polarization C-band data in with a window size of 13 and sigma value of 0.5.

**Figure 19:**
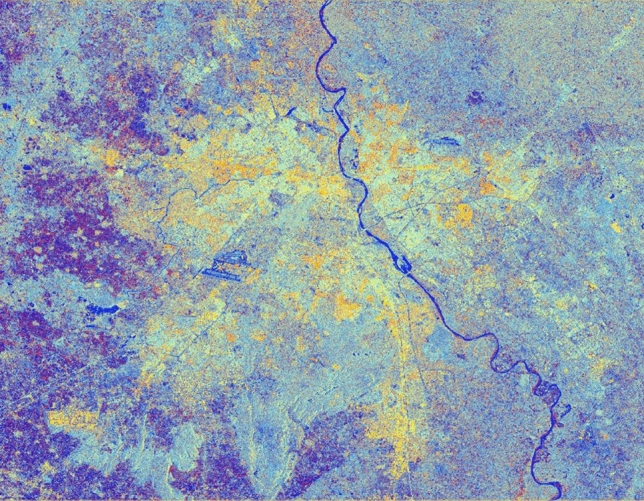
Colour composite image (Red: VV, Green: VH, Blue: VH/VV) of the Sentinel-1 dual-polarization C-band data in with a window size of 15 and sigma value of 0.5.

**Figure 20:**
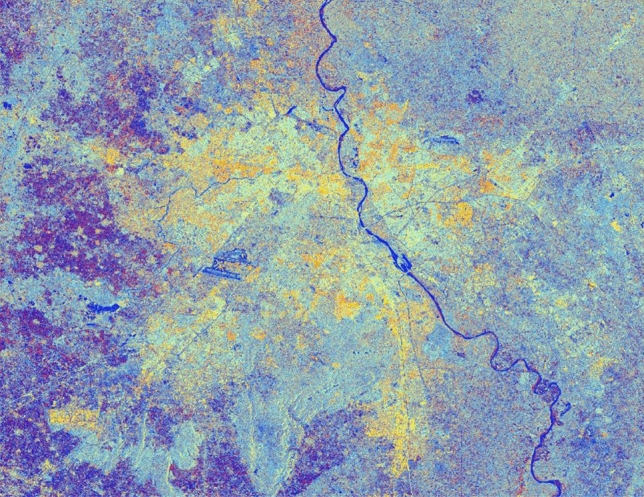
Colour composite image (Red: VV, Green: VH, Blue: VH/VV) of the Sentinel-1 dual-polarization C-band data in with a window size of 17 and sigma value 0.5.

The advancement of SAR technology facilitates the use of dual-polarization data for a better and efficient application for various societal purposes but as these datasets are effected due to speckle noise, hence suitable speckle filtering is required to perform before any application. So, in principle, filtering should be applied to enhance the features and suppress the surrounding noise. The simulated results are shown in Figs. 15, 16, 17, 18, 19, 20 exhibits the effect of these speckle algorithms to discuss several important issues related to the speckle filtering approach. All the figures are being generated with the change in filter properties for a constant sigma value of 0.5.

#### Simulated C-band SAR Image with a window size of 9 and sigma value of 0.5

See Fig. 16.

#### Simulated C-band SAR Image with a window size of 11 and sigma value of 0.5

See Fig. 17.

#### Simulated C-band SAR Image with a window size of 13 and sigma value of 0.5

See Fig. 18.

#### Simulated C-band SAR Image with a window size of 15 and sigma value of 0.5

See Fig. 19.

#### Simulated C-band SAR Image with a window size of 17 and sigma value of 0.5

The influences of the different window sizes in the generation of the colour composite (CC) images can be seen in Figs. 15, 16, 17, 18, 19, 20, which are very much useful in the interpretation of the various types of urban features. Similarly, Tables 1, 2, 3, 4, 5, 6, 7, 8, 9 summarizes the effect of filter properties on statistical variation in the image. These tables report the statistical summary of $\upsigma$0 values in different bands. These generated results also indicate that there is also an influence of window sizes such as 5, 11, 13, 15, and 17 with a sigma value of 0.5. But there is no variation in their statistical values. Almost all the statistical values for all the composite images shown in Figs. 15, 16, 17, 18, 19, 20 remains the same. The similar types of procedures can be applied with other sigma values like 0.6, 0.7, 0.8, and 0.9 with multiple groupings of diverse window sizes for understanding the influence of the window size and sigma values in image enhancement and speckle removal, which may, in turn, will be helpful in the identification of urban objects/ features for detailed or precise justifications.

**Table 2:** Statistical summary of $\upsigma {}^{0\ }$values in different bands.

	R	G	B
Unit	Int${}_{dB}$	Int${}_{dB}$	Nil
Min	−36.915	−35.788	−20.386
Max	15.955	13.627	167.658

**Table 3:** Statistical summary of $\upsigma {}^{0\ }$values in different bands.

	R	G	B
Unit	Int${}_{dB}$	Int${}_{dB}$	Nil
Min	−36.915	−35.788	−20.386
Max	15.955	13.627	167.658

**Table 4:** Statistical summary of $\upsigma {}^{0\ }$values in different bands.

	R	G	B
Unit	Int${}_{dB}$	Int${}_{dB}$	Nil
Min	−36.915	−35.788	−20.386
Max	15.955	13.627	167.658

**Table 5:** Statistical summary of $\upsigma {}^{0\ }$values in different bands.

	R	G	B
Unit	Int${}_{dB}$	Int${}_{dB}$	Nil
Min	−36.915	−35.788	−20.386
Max	15.955	13.627	167.658

**Table 6:** Statistical summary of $\upsigma {}^{0\ }$values in different bands.

	R	G	B
Unit	Int${}_{dB}$	Int${}_{dB}$	Nil
Min	−36.915	−35.788	−20.386
Max	15.955	13.627	167.658

**Table 7:** Statistical summary of $\upsigma {}^{0\ }$values in different bands.

	R	G	B
Unit	Int${}_{dB}$	Int${}_{dB}$	Nil
Min	−36.915	−35.788	−20.386
Max	15.955	13.627	167.658

**Table 8:** Statistical summary of $\upsigma {}^{0\ }$values in different bands.

	R	G	B
Unit	Int${}_{dB}$	Int${}_{dB}$	Nil
Min	−36.915	−35.788	−20.386
Max	15.955	13.627	167.658

**Table 9:** Statistical summary of $\upsigma {}^{0\ }$values in different bands.

	R	G	B
Unit	Int${}_{dB}$	Int${}_{dB}$	Nil
Min	−36.915	−35.788	−20.386
Max	15.955	13.627	167.658

### Selection of suitable filter for urban feature identification from C-band SAR Image

The technique of preserve and detect the spatial features with filtering operator authenticates the strength of a particular and it dependent on statistical values like mean, standard deviation and size of the filter window or mask. In various filters, the central pixel is not processed but in some of the filters do process the central pixel also along with the surrounding the pixel. Generally, the size of a window is expressed by the central pixel and the surrounding adjacent pixel based on the local statistics. Many of the time, this property is being used for point target detection. In some of the cases before applying filtering operation to central pixel, we do check it for threshold, if the pixel crosses a particular threshold then it will be filtered or else it will not be processed. There are several filters available for the image filtering but in case of radar or SAR datasets, these typical filter fails due to the diverse properties of the targets. The speckle effect is typically caused by random constructive and destructive interference of the de-phased but coherent return waves within each resolution cell of the SAR image. The presence of speckle noise in SAR datasets reduces the quality of images and it works as a hurdle for better image interpretation. It is essential to either suppress or reduce the presence of speckle noise from the SAR image before any of the information extraction. Speckle or spatial filtering has been an active research area for many years, accompanying the research for dual polarimetric SAR data set has been an active research domain by several researchers. Hence, the adaptive-window based speckle filter is required to be tested before extracting any information from the SAR datasets. Some of the SAR image filtering approaches adopt different filtering windows in both shape and size, based on the homogeneity and gradient information in the available datasets. Since the required input parameters of this adaptive window-based approaches are easy to set, these filters are easy to use.

Research investigation on the SAR image to suppress or reduce the inherent salt and pepper-like texturing called speckles is performed with the help of speckle filter operators. These operators are capable to handle the speckle noise of various distributions (Gaussian, multiplicative or Gamma) and this operator includes Boxcar (mean), Median, Frost, Lee, Refined Lee, Gamma-MAP, Lee Sigma, IDAN. The processed image obtained after the range-Doppler terrain are further processed with different filter speckle filter operator to understand and identify the best suitable filter for urban studies. The SAR image properties in both polarisation bands (i.e. VH & VV) are summarised in tabular form to demonstrate that the application of adaptive-window based filters in speckle suppression, detail preservation, and polarimetric information preservation.

The application of Lee filter, Lee-Sigma filter, Refined-Lee filter, IDAN filter, Gamma Map filter, Frost filter is capable to reduce the grainy in appearances in the SAR image but only a few filters like Lee, Lee-sigma and Frost can preserve the edge information with more minute detail. The similar window size of 3x3 is applied in all cases. The comparison between Lee and Lee-Sigma filter operation shows that even though the results are not so much different but still the results from Lee Sigma is slightly better. Both of them preserves edges, but Lee Sigma filter resolves the issue of haziness in the image. Lee Sigma is provided with parameters like (Number of Looks: 1, Window Size: 5$\times$5, Sigma: 0.5, and Target window size: 3$\times$3) and Lee (with filter size: 3$\times$3) and others variations of the Lee/Lee Sigma filters. But the results show (as summarized in Table 10) that the results from Refined-Lee and Frost are providing better results. Hence, before the extracting information processing of SAR datasets, the corresponding results obtained from the Refine-Lee speckle filter operator is used to study the different urban features in the area.

**Table 10:** Statistical summary of $\upsigma 0{}^{\ }$values in different bands for different filters.

Unit	Dual-Pol sigma0 dB VV+VH RGB image			Uniform window size or mask of 3 for Different Types Filter
	R	G	B	
	Int${}_{dB}$	Int${}_{dB}$	Nil	
Min	−36.915	−35.788	−20.386	Orignal values without Speckle Filter
Max	15.955	13.627	167.658	
Min	−26.91	−29.099	−14.837	Lee Speckle Filter
Max	14.778	11.018	20.938	
Min	−36.915	−35.788	−20.386	Lee-Sigma Speckle Filter
Max	15.955	13.627	167.658	
Min	−36.915	−35.788	−19.768	Refined-Lee Speckle Filter
Max	15.030	12.011	61.346	
Min	−25.384	−27.366	−575.275	IDAN Speckle Filter
Max	11.358	10.065	26.558	
Min	−26.91	−29.099	−14.837	Gamma Map Speckle Filter
Max	14.030	11.018	204.103	
Min	−26.91	−29.099	−15.226	Frost Speckle Filter
Max	14.801	11.185	24.305	

**Figure 21:**
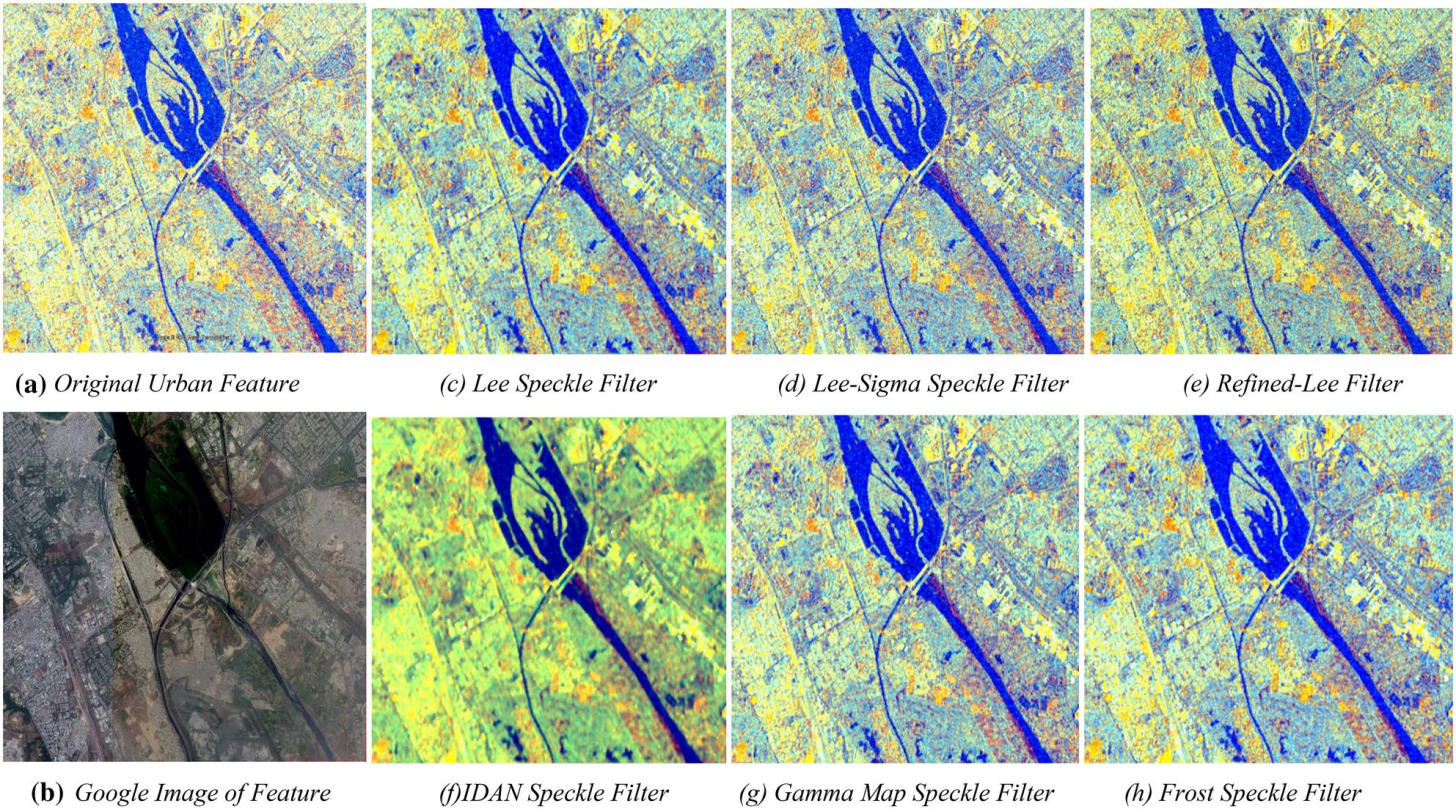
A detailed image of an urban area; (**a**) the original SAR image along with corresponding Google Image in (**b**) and the results of its filtration, using: (**c**) Lee filter, (**d**) Lee-Sigma filter, (**e**) Refined-Lee filter, (**f**) IDAN filter, (**g**) Gamma Map Filter, (**h**) Frost Filter; the size of window mask for all filters is 3.

Figure 21 in “Conclusion” shows the various output being generated from the processed SAR image after application of several filters including (c) Lee filter, (d) Lee-Sigma filter, (e) Refined-Lee filter, (f) IDAN filter, (g) Gamma Map Filter, (h) Frost Filter with a uniform window mask/ size of 3 for all filters. The output being generated are being presented in Fig. 21 in a tiled manner to give a comparative view of the different filter influences on the original SAR image. This is further used for selection of suitable filter for urban feature identification from C-band SAR image based on the capability of filter technique to preserve details and detect the spatial features with the help of statistical values like mean, standard deviation and size of the filter window or mask (as summarized in Table 10).

### Comparative assessment of filter for urban objects identifications from C-band SAR image

#### Identification/detection of urban objects with refined-Lee speckle filter

**Figure 22:**
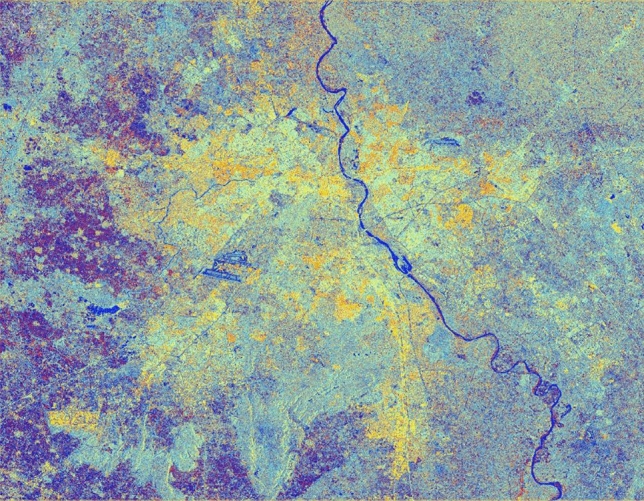
Dual-pol Sigma0 dB VV+VH Colour composite image (Red: VV, Green: VH, Blue: VH/VV) C-band Sentinel-1 image.

As mentioned in the earlier sections, dual-pol colour composite SAR images are being generated with the help of dual-pol processed datasets for better understanding and interpretation of the several urban features. Figure 22 shows the output image generated with the Refined-Lee Filter. It can be noted that all corresponding urban objects/ features are well highlighted as per their surfaces properties. The variation is coloured tone appears due to the type of urban feature and the composition of materials. The dry, arid or dehydrated features appear with high backscatter whereas wet or moist features appear as the low backscatter with lower colour tones. Table 11 presents the summary of Statistical summary of $\upsigma$0 values in different bands. The yellow colour appears in the scene highlights the presence of clustered pattern or clustered patch of urban settlements.

**Table 11:** Statistical summary of $\upsigma {}^{0\ }$values in different bands.

	R	G	B
Unit	Int${}_{dB}$	Int${}_{dB}$	Nil
Min	−36.915	−35.788	−19.768
Max	15.030	12.011	61.346

Similarly, the urban water bodies appear as low or dark tone and can be easily be identified from the radar images, as these features do not provide any backscatter to the radar sensors. Urban vegetation features or urban green cover appears with the medium backscatter values in the C-band radar images and these features can be easily seen around the urban settlements. The present investigation tries to report the response of radar signals from various urban features like urban settlements, urban water bodies *(including river, lakes, and ponds etc.)*, and man-made features* (like an airport runway, playground, and open green/open spaces)* in urban setups.

### Identification of sample site feature identification study

#### Analysis of urban water features

**Figure 23:**
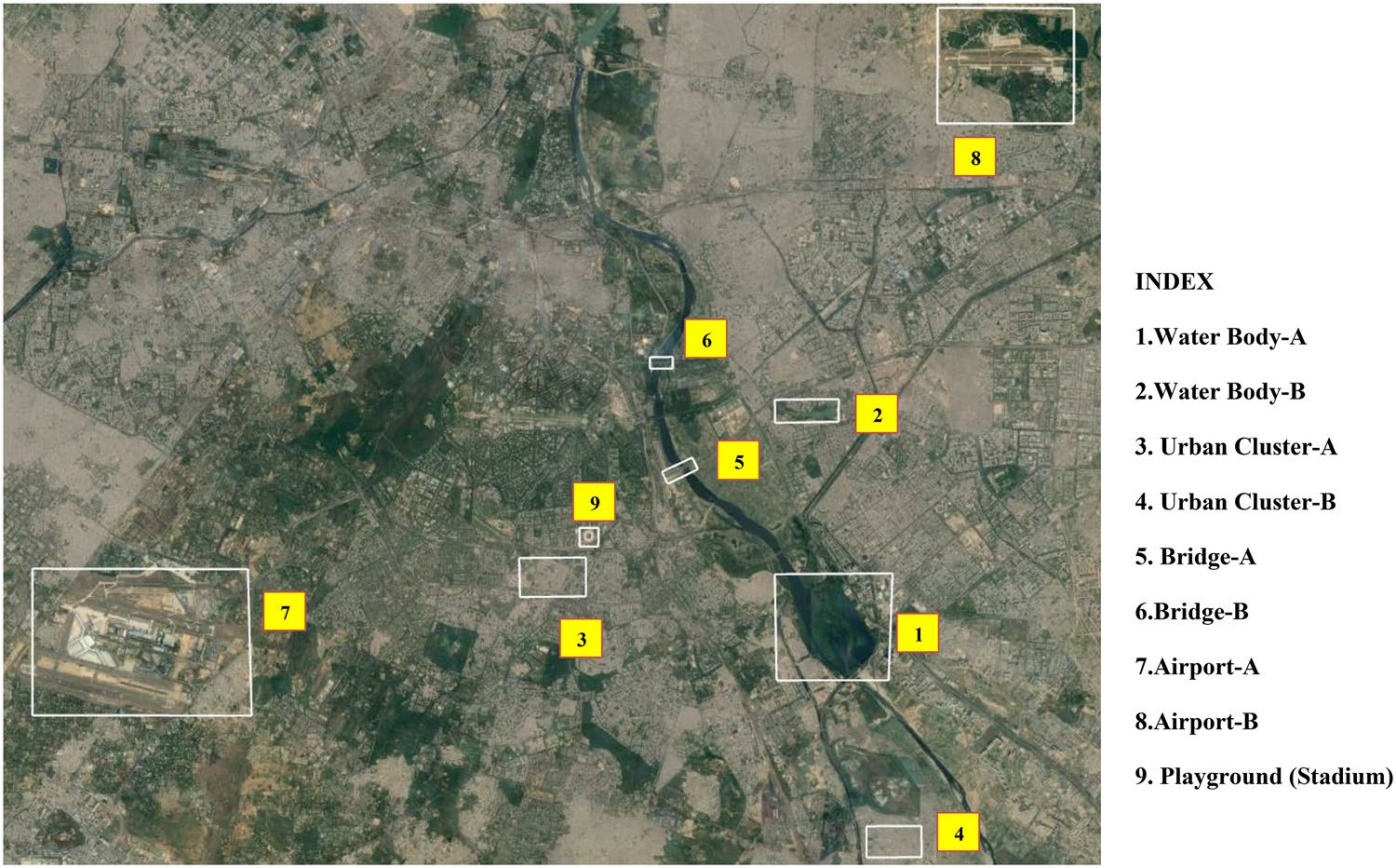
A detailed location of the sample site. Exhibits the sample selected locations for the detailed study of the urban features with the help of dual-polarised C-band SAR datasets.

Delhi is one of the biggest metropolitan regions in the northern part of India. The city is being traversed by the River Yamuna (sometimes it is also called as Jamuna). It is one of the largest tributary rivers of the Ganges (Ganga) in northern India and it flows through the religious land of Vrindavan. Figure 23 locate visualizes the spatial orientation and overview of the selected urban features from the study area. The river is being polluted with the release of toxic effluents from industries, the release of garbage and sewer water. It makes the water of the river as filthy and stinking. The city of Delhi typically experiences very hot and humid summers with a high temperature of around $47^\circ {\hbox {C}}$ in June to a low of $28^\circ {\hbox {C}}$ with a severe winter lowest temperature of $2^\circ {\hbox {C}}$. The city has an annual mean temperature of $25.3^\circ {\hbox {C}}$ (as per World Meteorological Department)^39^. The average altitude of the area ranges between 213 and 305 m and it covers an area of 1483 km${}^{2}$.

**Figure 24:**
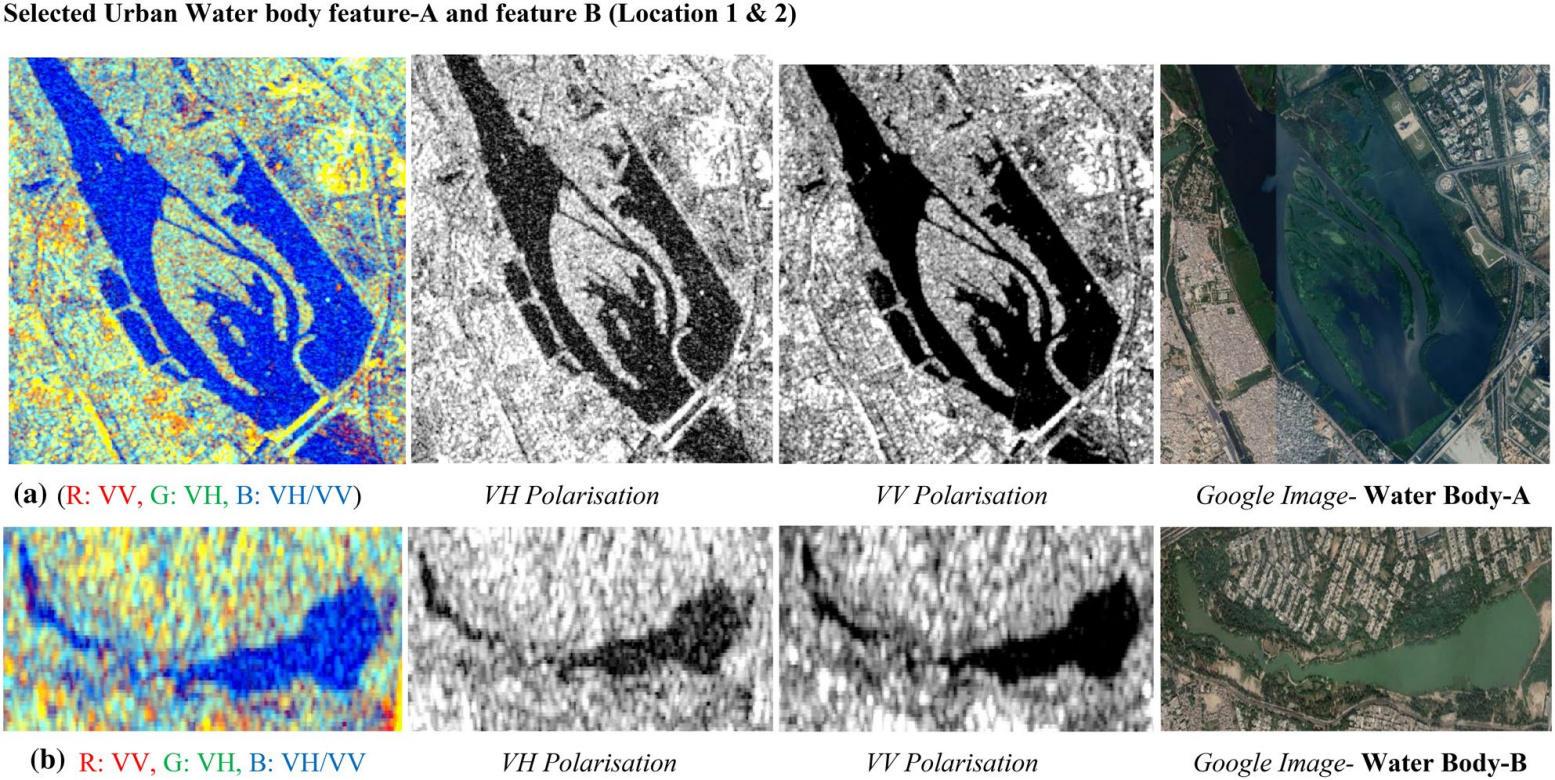
Colour composite image of the Sentinel-1 C-band data, dual-polarization data, and Google Image for site A & B.

Figure 24 displays the appearance of urban water features in both the polarization and the dual-polarised C-band colour composite SAR Image. Figure 24a displays dual-pol C-band colour composite SAR Image for Site A with individual polarised images, and corresponding Google Image for Site-A, similarly Fig. 24b displays dual-pol C-band colour composite SAR Image for Site B with individual polarised images, and corresponding Google Imagefor Site-B. Table 12 summarizes by the responses of radar backscatter values from urban water features/ waterbodies. It indicates the variation of backscatter values lies in the range of around −20 db for all water features for both the polarization. If we observe both polarization image with precise details then it can be observed that VH polarised image is more capable of identifying water feature sharper in compared to the VV polarised images. VV polarised sometimes assimilates soil or land or settlement feature/ area inside the water features, so VV polarization offers discrete detection capability for the surfaces. Whereas, VH image polarised images provide better detection of water features with backscatter values more than −20 db.Urban water features provide little response in VH polarizations in compared to VV polarizations and the same was observed at both selected sites with minimum backscatter value of −15.522 dB and maximum backscatter value of −29.048 dB in VV polarization along with minimum backscatter value of −28.295 in VH polarization, and maximum backscatter value of −17.270 dB in VH polarization.

**Table 12:** Summary of variations in backscatter values for urban water features.

Urban feature	Backscatter values (dB) in VV polarisation		Backscatter Values (dB) in VH polarisation	
	Minimum	Maximum	Minimum	Maximum
Waterbody-A	−29.048	−15.522	−28.295	−17.270
Waterbody- B	−29.049	−16.108	−27.149	−19.445

Selected Urban Water body feature-A and feature B (Location 1 & 2).

1. It can be perceived from Fig. 24a,b image that water features are well highlighted in VV polarisation in compared to VH polarisation. Investigation of urban settlement cluster from C-band SAR Image

**Figure 25:**
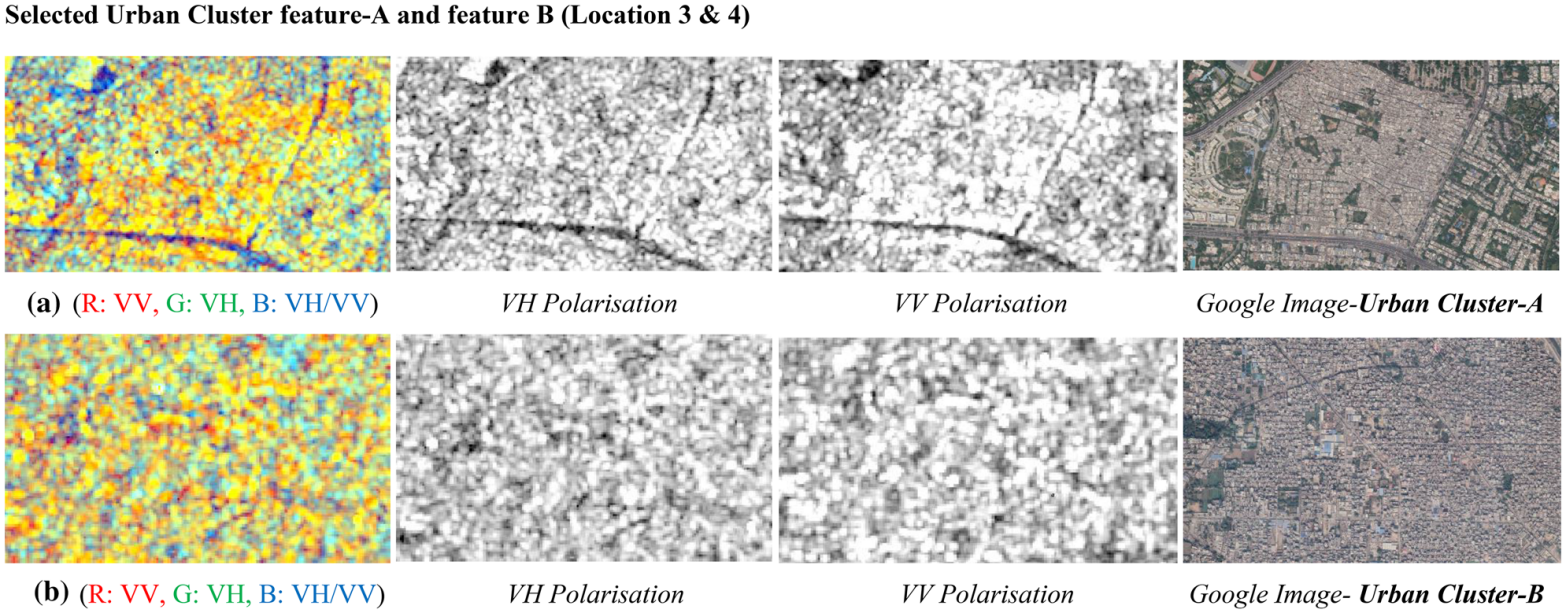
Colour composite image of the Sentinel-1 C-band data, dual-polarization data, and Google Image for site A & B.

Figure 25 illustrates the urban settlement features in both the polarization and a dual-pol C-band colour composite SAR Image with corresponding google earth image for visual validation.

The responses of backscatter return from different urban settlement clusters are being described in Table 13 with a summary statistics of backscatter values for both polarizations. It can be perceived that the minimum backscatter at cluster A is −18.247 dB in VV polarisation and −27.293 dB in VH polarisation, and maximum backscatter in VV polarisation is −10.587 dB and VH polarisation has −18.891 dB, which indicates that the backscatter values lie around −10 db in both the polarization. It can be observed that the VV polarised image is more capable of recognizing the urban settlement feature in compared to VV polarised images. It provides competence for optimal interpretation of the urban features for better understanding of the area.

**Table 13:** Summary of variations in backscatter values for urban settlement features.

Urban feature	Backscatter values (dB) in VV polarisation		Backscatter values (dB) in VH polarisation	
	Minimum	Maximum	Minimum	Maximum
Urban Cluster-A	−18.247	−10.587	−27.293	−18.891
Urban Cluster-B	−12.638	−5.355	−13.756	−6.466

Figure 25a displays dual-pol C-band colour composite SAR Image for Site A with individual polarised images along with corresponding Google Earth images for the Site-A, similarly Fig. 25b displays dual-pol C-band colour composite SAR Image for Site B with individual polarised images, and corresponding Google Imagefor Site-B respectively. The dry features in the radar backscatter images display the high backscatter values compared to values from wet or moist features. The low backscatter values infer that backscatter returns are from wet surface features and vice-versa. The tone of the images also appears to be highlighted due to the presence or absence of moisture content on the surface features. It can be perceived in the dual-pol C-band colour composite SAR Image that all the urban objects/ features are well highlighted with variation in their tone as per their surfaces properties of each feature for better understanding about the composition of the surface materials.

### Investigation of bridge & airport feature (man-made features) from C-band SAR image

#### Interpretation for bridges

**Figure 26:**
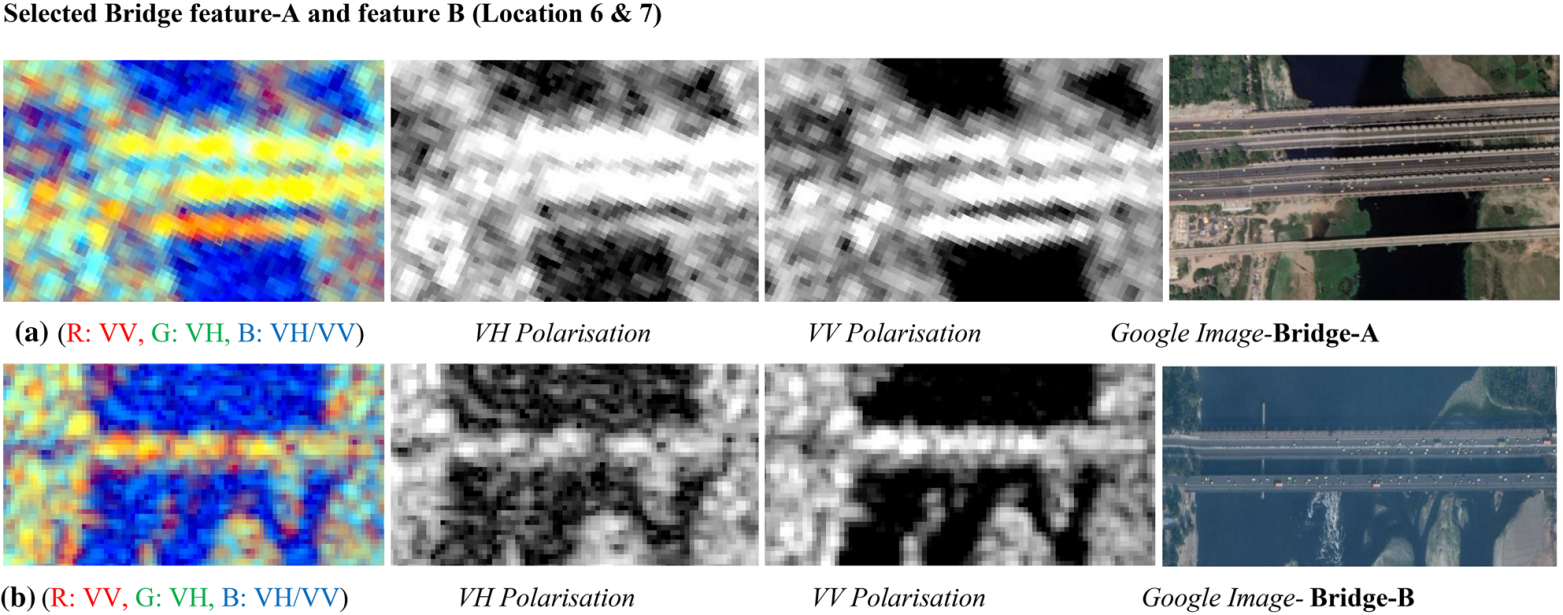
Colour composite image of the Sentinel-1 C-band data, dual-polarization data, and Google Image for site A & B.

It can be seen in Fig. 26 shows that bridges are prominently visible in the radar image and can easily be identified due to the presence of high backscatter returns/responses from the metallic or concrete structure. It is a well-known phenomenon that steel structure or urban features/objects shows the double bounce backscatters in the radar images and it causes brighter appearance in the image. The summary in Table 14 reports for radar backscatter responses from metallic bridges for both polarizations. VH polarization shows values of −7.336 dB and VV polarization shows the values of −2.611 dB. It shows the majority of values lies within the range of around 0 dB. The dual-pol C-band colour composite SAR Image is shown in Fig. 26a,b along with the backscatter responses for both polarizations and all metallic or concrete features are prominently highlighted as per their surfaces properties with brighter appearances in the corresponding SAR as per their material composition.

**Table 14:** Summary of variations in backscatter values for bridges.

Urban feature	Backscatter values (dB) in VV polarisation		Backscatter values (dB) in VH polarisation	
	Minimum	Maximum	Minimum	Maximum
Bridge-A	−4.674	−2.611	−7.295	−7.336
Bridge-B	−6.231	−3.337	−13.393	−10.545

Figure 26a presents dual-pol C-band colour composite SAR Image for Site A (Bridge) with individual polarised images, and corresponding Google Image for Site-A, Similarly Fig. 26b displays dual-pol C-band colour composite SAR Image for Site B (Airport Runway) with individual polarised images, and corresponding Google Image for Site-B. The bridge structure or feature for Site-A is prominently visible due to higher backscatter returns from the objects in the VV polarization image, as typically man-made feature exhibits the high backscatter returns.

The bridge structure in C-band dual-pol SAR image is seen as a linear structure with a brighter tone. Likewise, airport runway structure or feature for Site-B is visible in dark tone due to low backscatter responses from the objects in either of the polarization due to the specular surface or the smooth surface of the runway. Even in colour composite SAR Image, the airport runway structure is being seen with a dark tone and the same can be identified in the VV polarised SAR image.

#### Interpretation for airport runways

**Figure 27:**
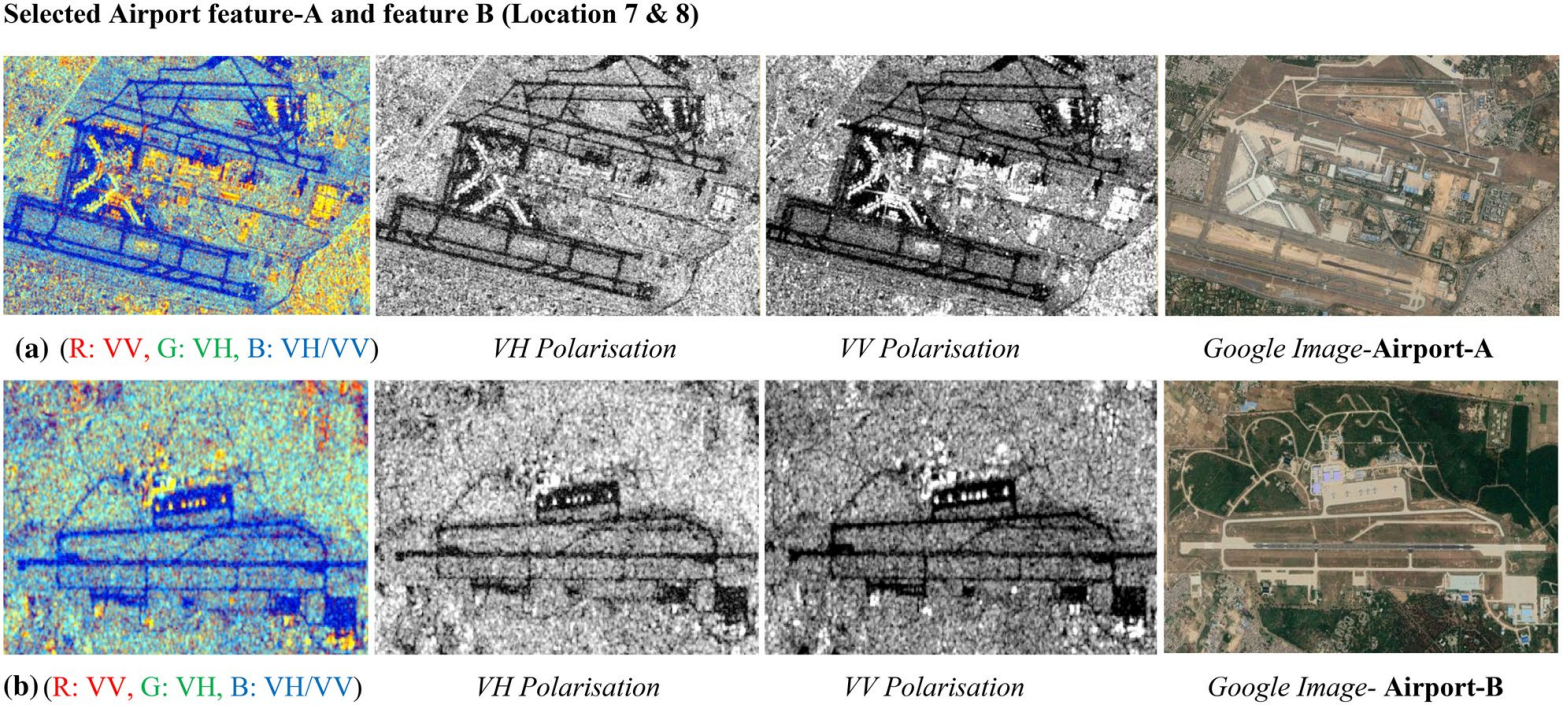
Colour composite image of the Sentinel-1 C-band data, dual-polarization data, and Google Image for site A & B.

It can be seen in Fig. 27 shows that airport runways along with the surrounding features including airport area can be easily identified due to the presence of high backscatter returns/responses from the metallic or concrete structure and no backscatters from the runway. It is a well-known phenomenon that steel structure exhibits the double bounce backscatters in the radar images, which causes brighter appearance in the image. The summary in Table 15 reports for radar backscatter responses from metallic bridges for both polarizations. VH polarization shows values of 9.63 dB and VV polarization shows the values of 3.926 dB from Airport-A. It shows the majority of values lies above 0 dB. The corresponding dual-pol C-band colour composite SAR images are also shown in Fig. 27a,b along with their backscatter responses. The presence of metallic objects and concrete features can be easily identified due to their brighter appearances as per the composition of materials.

**Table 15:** Summary of variations in backscatter values for bridges and airport runway.

Urban feature	Backscatter values (dB) in VV polarisation		Backscatter values (dB) in VH polarisation	
	Minimum	Maximum	Minimum	Maximum
Airport-A	−16.265	9.693	−23.542	3.926
Airport-B	−18.911	−1.221	−23.057	−5.355

**Figure 28:**
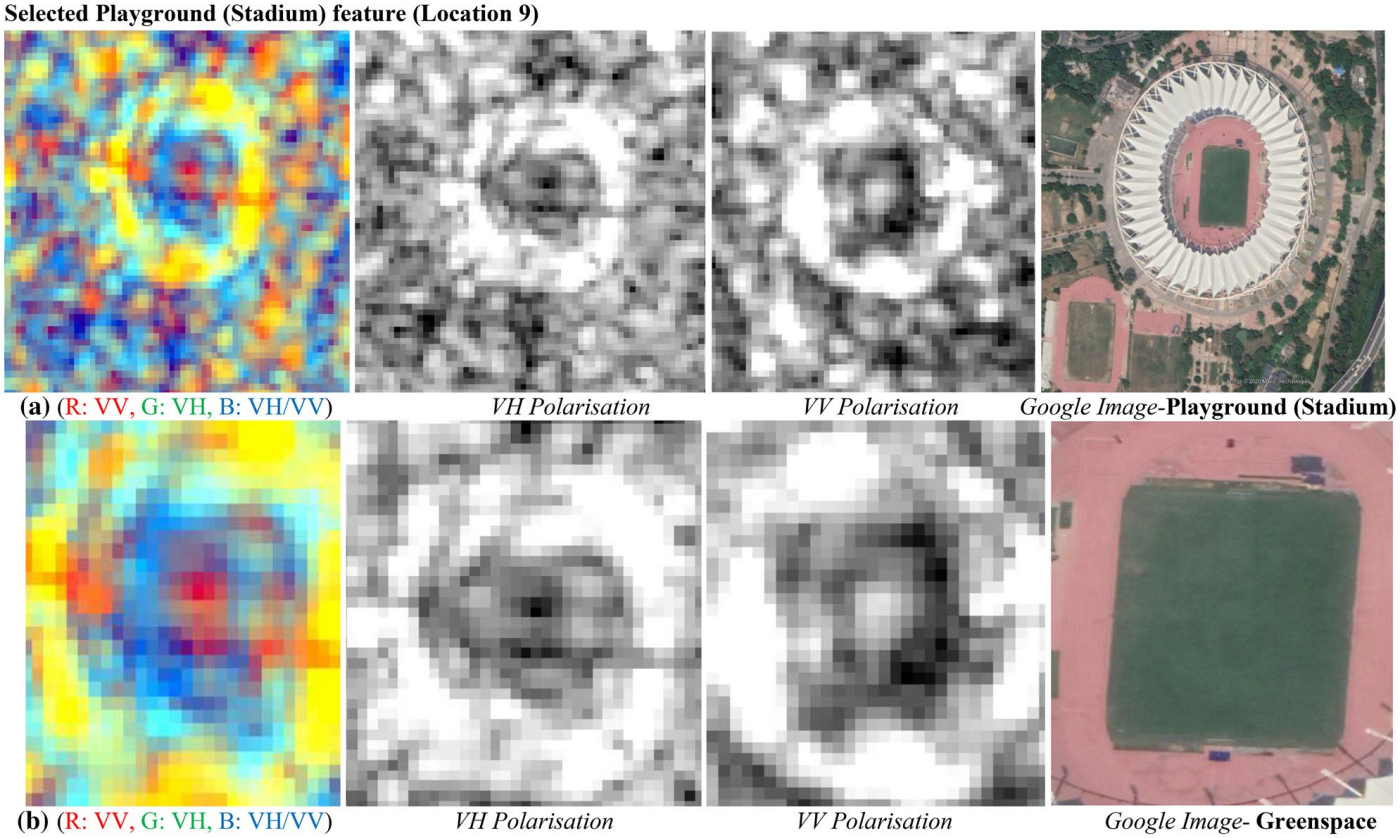
Colour composite image of the Sentinel-1 C-band data, dual-polarization data, and Google Image for site **a** & **b**.

Figure 27a presents dual-pol C-band colour composite SAR Image for Airport-A with individual polarised images, and corresponding Google image the same, Similarly Fig. 28b displays dual-pol C-band colour composite SAR Image for Airport-B with individual polarised images, and corresponding Google image. The complete airport structure for both the sites is prominently visible due to higher backscatter returns from the objects in the VV polarization image, as typically man-made feature exhibits the high backscatter returns. Airport runway structure for both the sites is seen in dark colour tone due to low backscatter responses in either of the polarization due to the specular surface or the smooth surface of the runways. Even in colour composite SAR Image, the airport runway structure is being seen as a dark feature and the same can be identified in the VV polarised SAR image.

#### Interpretation for playground (stadium) and greenspace

It can be perceived in Fig. 28 that green space is identifiable due to the better responses of the radar backscatter signals from green cover. It occurs due to the volumetric scattering mechanism. It can be seen that VH polarised images exhibit the clear and exact orientation of urban greenspace feature in the processed image. It is a well-known phenomenon that wet features are well interpreted with VV polarised images due to presence water and the same concepts is vice-versa for man-made or dry features like a playground. Due to smoother surface of playground, it behaves like perfect specular surface and consequently the backscatter signals at the SAR sensors is obstructed in VH polarization and it exhibits values more than −1.294 dB and lower values below than −6.488 dB in VV polarization.

Table 16 describes the backscatter values for both the sites in their corresponding individual polarization. It can be perceived minimum backscatter value is −6.488 dB in VV Polarisation with minimum backscatter value is −17.974 dB in VH polarisation, and maximum backscatter value is −1.294 dB in VV polarisation and maximum backscatter value is −10.687 dB in VH polarisation. It infers that variation in values lies less than −20 dB in both the polarizations for the mentioned features.

**Table 16:** Summary of variations in backscatter values.

Urban feature	Backscatter values (dB) in VV polarisation		Backscatter values (dB) in VH polarisation	
	Minimum	Maximum	Minimum	Maximum
Playground (Stadium)	−6.488	−1.294	−17.974	−10.687
Green space	−10.895	−4.495	−28.030	−13.748

Figure 28a displays dual-pol C-band colour composite SAR Image for Playground (Stadium) with individual polarised images, and corresponding Google image. Similarly, Fig. 28b displays dual-pol C-band colour composite SAR Image for Greenspace with individual polarised images, and corresponding Google image.

## Conclusion

C-band synthetic aperture radar (SAR) images acquired from Sentinel-1A/B satellite offers free dual-polarization VV *(Vertical transmit Vertical receive)*, VH *(Vertical transmit Horizontal receive)* and has different configurations, resolutions, band combinations in their corresponding ascending and descending orbits. Since radar data requires several specific algorithms to realise calibrated and orthorectified images, therefore the work attempted for the pre-processing of Sentinel-1 datasets with the help of open-source SNAP toolbox to propose a comprehensive robust methodology with validation for the speckle noise removal or suppression from the dual-polarized SAR datasets through the application of spatial or speckle filters. The present approach acknowledged Lee or Lee sigma filters are capable preserve the details of edges information with noise reduction but Refined –Lee filter works well with urban object identification and detection. It also showcases the approach for identification or detection of urban features of objects *(like urban settlement, urban water bodies, runways, bridge, playgrounds, and urban open space/green cover including man-made objects)* from freely available C-band dual-polarized datasets with the help of spatial (speckle) filters. It presents a distinct evaluation of urban object/ feature detection/identification approaches with speckle filters at pixel-level to understand the performance of each approaches with a set of dual-pol SAR images and corresponding Google Maps (*for feature identification and labelling)* images for ground truth verification. The experimental results revealed that the proposed technique works well over traditional methods in terms of accuracy and precision. The work can be extended further with the variations in the filter parameters and can also be expanded further to apply the speckle filters on every cloudy pixel to generate noiseless images. In addition to this, more results for different seasons can be generated for other urban objects with these models for further improvement of approach, which will ascertain the performance and sensitivity for other topographic areas.
